# Ensemble Effects on Hydroxide Bond Dissociation Free Energies in Polyoxovanadate Clusters

**DOI:** 10.1021/acs.jpca.5c04885

**Published:** 2026-02-09

**Authors:** Andreas Towarnicky, John N. El Berch, Giannis Mpourmpakis

**Affiliations:** † Department of Chemical Engineering, 6614 University of Pittsburgh, Pittsburgh, Pennsylvania 15261, United States; ‡ School of Chemical Engineering, National Technical University of Athens (NTUA), Athens, GR 15780, Greece

## Abstract

Understanding structure-property relationships is foundational to numerous modern chemistries, such as proton-coupled electron transfer (PCET). However, an experimentally measured property is the result of the behavior from an ensemble of molecules. Neglecting ensemble effects, especially under complex chemical environments, may obfuscate these relationships and lead to discrepancies between theory and experiment. In this work, we demonstrate the impact of configurational entropy and local chemical environments on hydroxide bond dissociation free energies [BDFE­(O–H)] for a set of polyoxovanadate nanoclusters, at ambient conditions. The O–H bond strengths are investigated via density functional theory (DFT) coupled with statistical thermodynamic analysis and bilinear modeling, and compared with previous experimental results on the same systems, namely electrochemical solutions of: [V_6_O_13–*x*_(OH)_*x*_(TRIOL^R^)_2_]^−2^ (*x* = 2, 4, 6; R = NO_2_, Me) and [V_6_O_11–*x*_(OMe)_2_(OH)_*x*_(TRIOL^NO_2_^)_2_]^−2^ (*x* = 2, 4). Interestingly, we find that ensemble effects, even at room temperature, can account for a significant portion of the BDFE­(O–H) trend with the degree of reduction via H atom binding, which cannot be fully captured by single-structure, static DFT calculations. Moreover, we find that the ensemble effects may be replicated statistically, requiring only enumeration of energetically accessible H-binding sites. With the ensemble effects resolved, we present a simple bilinear model to reconcile remaining biases between experiment and ensemble-informed theory, which corelate with cluster-specific electronic environment differences. The bilinear model achieves outstanding accuracy vs experiments with a root-mean squared error of 0.4 kcal/mol. Finally, based on the physicochemical characteristics of hydrogen interaction with polyoxometalates, we present a simple methodology that captures the BDFE­(O–H) trend while dramatically reducing required DFT calculations by 98% and achieving accuracy within 1 kcal/mol. Overall, this work elucidates the roles and structural origins of configurational entropy and chemical effects on polyoxometalate hydroxide bond energies, with potential applicability to various atomically precise metal oxide systems. Importantly, it introduces models for rapid and highly accurate property calculations in connection with experiments.

## Graphical abstract

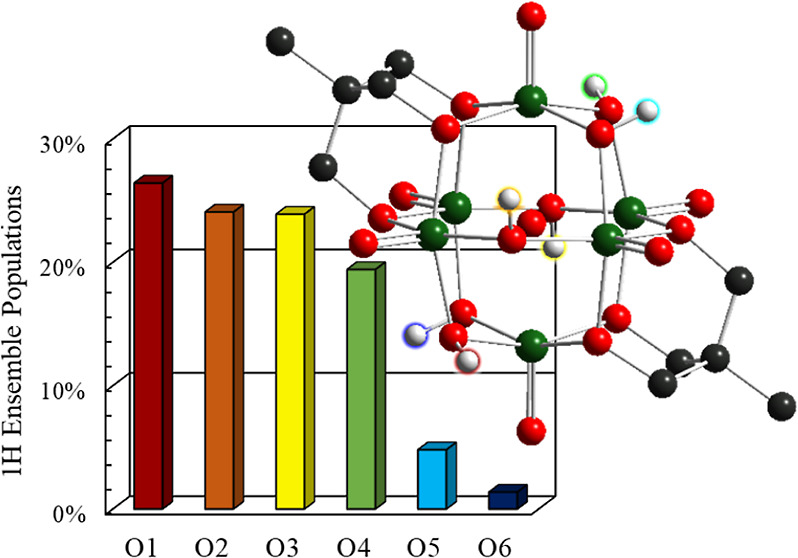

## 1 Introduction

Advancing modern technologies requires fundamental understanding of complex phenomena, including PCET,^1,2^ and their manifestations in gainful systems such as metal oxides^3^ and polyoxometallates (POMs).^4–6^ PCET is equivalent to hydrogen atom transfer (HAT) when colocated and concerted,^7,8^ and by the natural ubiquity of hydrogen, is thus central to a large number of chemical reactions^9–13^ as well as their catalysis.^7,14,15^ Although fundamental, challenges remain to elucidating PCET at ensemble scales,^16,17^ including significant computational expense and lingering gaps between theory and experiment. Metal oxides participate in many PCET applications, and offer the ability to tailor PCET mechanisms^18^ while providing versatility, structural stability, and tunable^19^ physicochemical properties. POMs are atomically precise metal oxide clusters with similar surface bonding motifs to extended metal oxides, thus, bridging molecular to extended systems.^20,21^ These advantages make POMs ideal systems to investigate PCET chemistries, improve understandings, and hone computational approaches.

A key descriptor of PCET is the bond dissociation free energy,^22–25^ and specifically with POMs, the hydroxide BDFE­(O–H).^26,27^ BDFE­(O–H) values describe the homolytic cleavage of an H atom, and are thermodynamically equivalent to successive electron and proton transfers, in either order, which comprise the overall reaction.^7,25^ Therefore, changes in the electron affinity or basicity of POMs can influence the BDFE­(O–H), which further connects to PCET kinetics via Marcus theory.^23,27,28^ Understanding of the various influences on BDFE­(O–H) and thus PCET, is central to electrocatalytic chemistries such as the oxygen reduction reaction (ORR).^26^

Previous investigations have provided substantial understanding of various aspects of POM chemistries. Work by Maeda et al. determined that more negative POM cluster charges effect more negative electrochemical reduction potentials^29^ (less exergonic electron affinities). Changes in electron affinity translate directly to changes in BDFE­(O–H) if the proton transfer energetics remain the same, and have been shown to manifest in POM-catalyzed chemistries.^30^ Similarly, changes to POM constituent metals were demonstrated to affect POM reduction potentials^19,31–33^ and BDFE­(O–H) in alignment with the energetics of reducing the metal centers.^34^ Different POM morphologies were previously shown to influence different site-specific basicities,^35^ which were revealed to extend to morphology-dependent and site-specific BDFE­(O–H).^36^ Solvation effects on small molecule BDFE­(O–H) have generally been found to be minor,^37,38^ but can be substantial with POMs if specific interactions contribute to coordination-induced bond weakening.^39^ Functionalization of POMs with different ligands has been illustrated to modify their reduction potentials.^40^ In our recent work we demonstrated that changes in ligand identity (Figure 1), such as their electron-withdrawing or donating nature, can change POMs electron affinity.^41^ Although this effect may be largely offset in their impact on BDFE­(O–H) by concomitant changes in basicity,^41^ a phenomena known as thermodynamic compensation. Additionally, this work further demonstrated an increase in electronic communication across the Lindqvist core due to surface methylation,^40,41^ and that BDFE­(O–H) was strongly influenced by the charge transfer to the bridging oxygen atoms.^41^ However, a moderate degree of offset was present between the experimental and computationally derived BDFE­(O–H) values (Figure 2b, *vide infra*), and the changes in BDFE­(O–H) effected by successive H atom reductions were incompletely captured by theory.^41^

**Figure 1:**
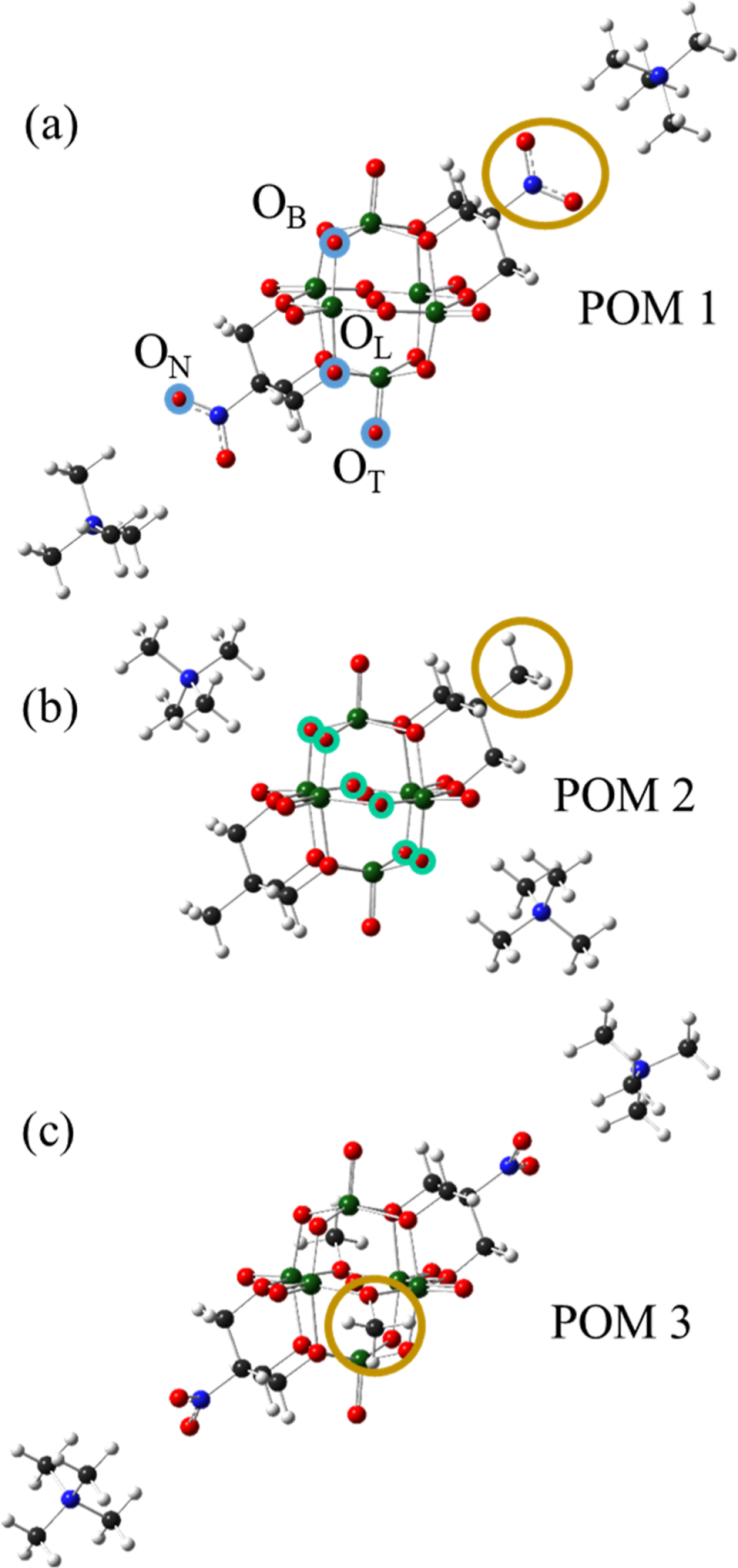
DFT geometrically optimized structures of: (a) [Me_4_N]_2_[V_6_O_13_(TRIOL^NO_2_^)_2_] (POM 1), (b) [Me_4_N]_2_[V_6_O_13_(TRIOL^Me^)_2_] (POM 2), and (c) [Me_4_N]_2_[V_6_O_11_(OMe)_2_(TRIOL^NO2^)_2_] (POM 3). Functionalization of POM 1 with two surface methyl groups results in POM 3 in (c). Atom Key (spheres): green, V; red, O; black, C; blue, N; white, H. Brown circles indicate different functionalization of POMs: nitro-functionalized TRIOL ligands in POM 1, methyl-functionalized TRIOL ligands in POM 2 and surface methylation in POM 3. Blue circles on POM 1 indicate different possible H-binding oxygen sites, Bridge (O_B_), Terminal (O_T_), Ligand (O_L_), and Nitro (O_N_); the center internal oxygen is not surface accessible. Green circles on POM 2 indicate the six thermodynamically favored O_B_–H binding sites (analogous to the 6 O_B_ of POM 1). POM 3 has only 4 O_B_ atoms available.

**Figure 2:**
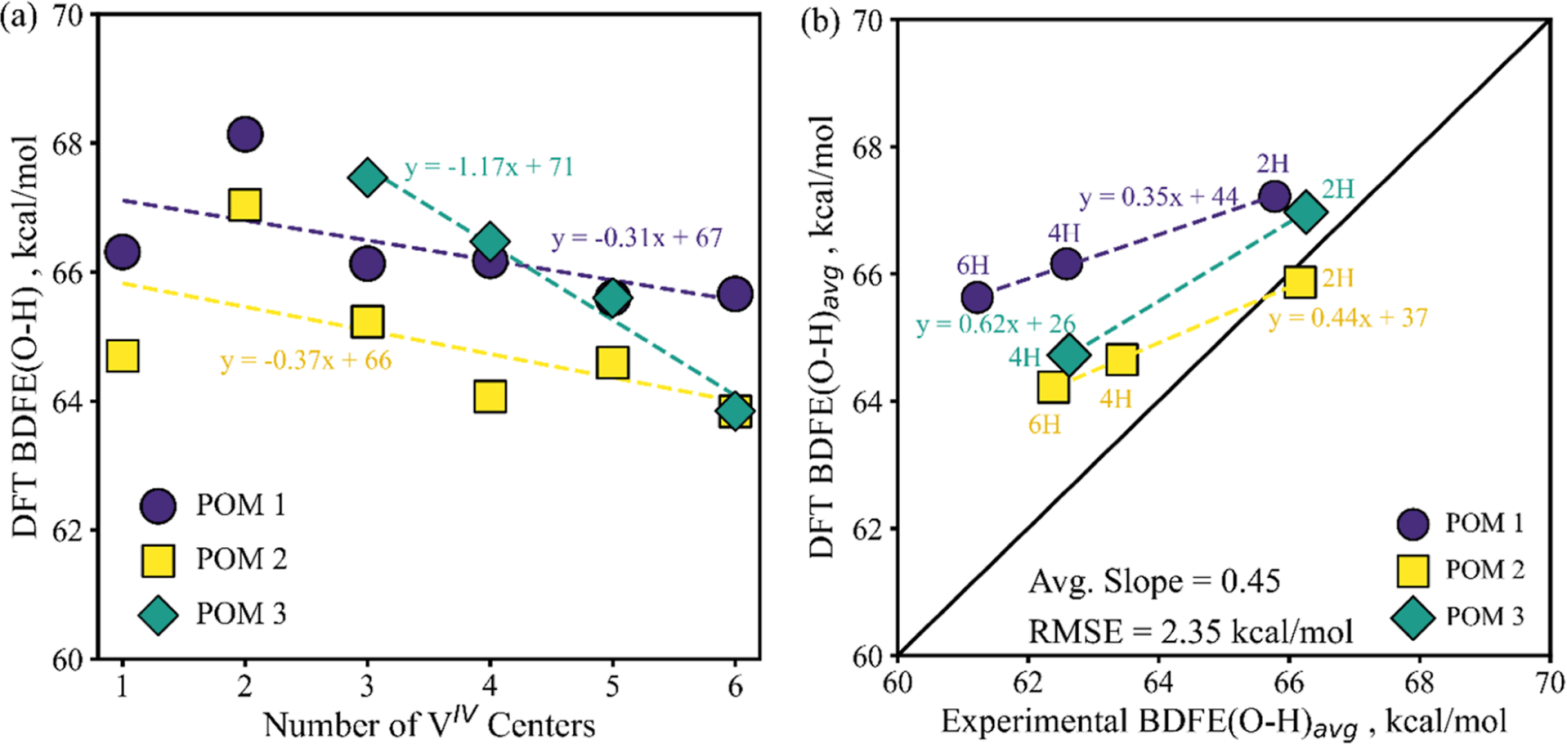
(a) DFT BDFE­(O–H)_*x*_ of each POM, where *x* = 1–6 for POMs 1 and 2, corresponding to the same number of V^IV^ centers, and *x* = 1–4 for POM 3, corresponding to 3–6 V^IV^ centers; and (b) parity plot of DFT vs experimental BDFE­(O–H)_avg_. The POM trends of (a) are plotted vs the number of V^IV^ centers to show the values at the same cluster oxidation states. These represent the same data published in our previous work with the Matson group.^41^

Improved understanding is key to close residual theory-experiment gaps and enable greater accuracy and efficiency from first-principles calculations. To this end it is instructive to consider the atomically precise systems of our prior work^41^ in greater detail. Specifically, in collaboration with the Matson lab, we compared experimentally measured 2H atom average BDFE­(O–H)_avg_ with corresponding values calculated via DFT.^41^ DFT satisfactorily corroborated the experimentally observed trend of decreasing BDFE­(O–H) with increasing degree of H atom reduction to the POMs (see the BDFE­(O–H) slopes of Figure 2, *vide infra*), but DFT underestimated the trend by about half, and point-specific differences of 1 to 4 kcal/mol were present. The incomplete trend-capture result is similar to previous findings by Agarwal, Kim, and Mayer,^42^ who noted that the change in BDFE­(O–H) upon increasing H atom reduction of mixed valence ceria nanoparticles (Ce^3+^/Ce^4+^) could not be satisfactorily explained with Nernstian mass-action or Langmuir chemisorption models. In fact, on their 2–4 nm nanoparticles with on the order of 100 potential binding sites, they found that these models would underestimate the BDFE­(O–H) changes by an order of magnitude (more than 10 kcal/mol), highlighting the significance of the degree of metal center reduction by H atoms. In our POM systems,^41^ DFT provided improved trend-capture, attaining ∼50% compared to ∼20% by Nernstian analysis.^6^ However, the remaining BDFE­(O–H) differences between theory and experiments are sufficient to eclipse other effects (e.g., differences in ligation), and suggest that further theoretical consideration may be beneficial.

Two possible areas for greater examination are discerned from considering the nature of molecular DFT calculations and the experiments themselves. The experiments in our prior work^41^ were on electrochemical solutions, with millimolar POM concentration, organic acid, and buffers, and their BDFE­(O–H) values entail the measurement of POM interaction with the electrode surface and its boundary layers.^43^ In our previous investigations, DFT considered individual POM structures and thus did not account for ensemble effects that may be present in solution, particularly among energetically accessible POM isomers. The DFT calculations also do not account for how the complex chemical environment may affect POM BDFE­(O–H). Our prior work further illustrated a significant impact of the electronic environment on BDFE­(O–H) in relation to the POM bridging oxygen (O_B_) charges.^41^ We hypothesize that considering these aspects may help resolve gaps between experiment and theoretical calculations.

Initial inspection of the POM structures with regard to the number of possible POM O–H binding sites (Figure 1, vide infra) suggests that a configurational entropy factor *S* ^Config^ may be on the order of 1 to 2 kcal/mol per H binding, which is commensurate to the discrepancy between the computational and experimental BDFE­(O–H) trends. Considering insights from the hydrogenation of metal oxide extended surfaces, Fripiat and Lambert found that *S* ^Config^ and averaging across different site energies (affording a difference vs the minimum site energy, herein termed Δ*G* ^BA^) were key in fitting theory to experimental adsorption isotherms,^44^ and additionally that the *S* ^Config^ could be determined via sterically informed statistical thermodynamics. Furthermore, the *S* ^Config^ contribution to Langmuir adsorption isotherms is generally known^45^ and may be inferred to extend to chemisorption. Various studies by our group^46,47^ and others^48–50^ have shown how considering ensemble effects can improve the prediction of thermochemical properties and catalyst behavior. However, additional multiscale modeling efforts, such as incorporating MonteCarlo sampling, are often required to resolve *S* ^Config^ and ensemble effects,^51,52^ and their resolution via DFT may be impractical for combinatorially complex systems. Notwithstanding challenges via other methods, and noting the atomically precise POM structures and previous connection to O_B_ charges, we were motivated to explore whether ensemble effects and the electronic environment could be accounted for via statistical thermodynamics and linear incorporation of an atomic charge factor, respectively.

To the best of our knowledge, the role of ensemble effects in POM hydrogen transfer energetics has yet to be described, especially through fundamental statistical thermodynamics and structure-function relationships. In this work we demonstrate how the presence of multiple, near-equivalent hydrogen-binding sites on POMs give rise to ensemble effects on subnanoscale oxide systems that can affect the calculated POM property (herein, BDFE­(O–H)). Further, we demonstrate that accounting for the local chemical environment via bilinear modeling with O_B_ charges may close systemic offsets between theory and experiment. Overall, we introduce new computational avenues for rapid calculations of BDFE­(O–H) values, leading to improved accuracy in modeling POMs and similar systems.

## 2 Computational Methods

### 2.1 Density Functional Theory

DFT calculations were performed using the Gaussian 16 program package.^53^ The hybrid functional B3LYP^54^ was used together with the LANL2DZ basis set,^55^ which afforded computational efficiency while maintaining accuracy.^56^ Implicit solvation was utilized via the self-consistent reaction field with acetonitrile as solvent, corresponding to the experiments^41^ [SCRF = (SMD,acetonitrile)].^57^ This level of theory resulted in very good agreement between theory and experiments in describing BDFE­(O–H)_avg_ trends on polyoxovanadates^41^ and polyoxotungstates.^36^

DFT geometrically optimized structures with counterions are illustrated in Figure 1 for the same POMs as investigated experimentally.^41^ DFT calculations utilized tetramethylammonium counterions instead of the tetra­(*n*-butyl)­ammonium used in experiments^41^ to reduce computational cost. All structures were fully optimized to standard tolerances (RMS force ≤ 3 × 10^–4^ hartree per Bohr)^53^ or even tighter criteria. Vibrational frequencies were computed for the optimized geometries.^58,59^ confirming the energy minima to be free of imaginary frequencies. The DFT-optimized structures matched those previously resolved via XRD with great agreement.^41^ Thermochemical values were determined at 298.15 K and 1 atm,^58,59^ corresponding to experimental conditions.^41^ Natural Bond Orbital (NBO)^60^ analysis was used to calculate atomic charges. Atomic visualizations were developed with GaussView 6.^61^

Gibbs free energy values were used to compare relative energetics of different configurations and spin states. Elevated spin states were evaluated for each hydrogen binding configuration investigated, up to a multiplicity corresponding to (#V^IV^ + 2) unpaired electrons, where #V^IV^ is the number of reduced vanadium atoms (e.g., [V_6_O_9_(OCH_3_)_2_(OH)_2_(TRIOL^NO2^)_2_]^−2^ has 4 V^IV^ centers and all spin states were investigated up to a multiplicity of septet). Beyond the multiplicity corresponding to #V^IV^ unpaired electrons, a significant increase in calculated energy was invariably observed (Figure S1). In these structures, no significant spin contamination was noted. For each degree of H atom reduction, the lowest energy combination of spin state and H-binding configuration was utilized for BDFE­(O–H) calculations.

To find the lowest energy DFT structures, for each degree of H-binding we considered incremental configurations (adding 1H at a time) to each of the available oxygen atoms, i.e. considering at least one incremental configuration for all Bridge, Terminal, Ligand, and Nitro oxygen (O_B_, O_T_, O_L_, and O_N_, respectively) (Figure 1a, blue circles). For each 1H increment, the lowest energy configuration was retained for the next 1H evaluation. Two orientations of O–H dihedral angle are possible for each O_B_–H binding (Figure S2a). For POMs 1 and 2, considering all possible combinations with both O_B_–H configurations becomes computationally prohibitive, requiring at least 2005 DFT geometry optimizations and vibrational analyzes for each POM (Table S1). Thus, for POMs 1 and 2, only O_B_–H combinations with the same direction of O=V–O–H dihedral angle were considered (i.e., all configurations with all O–H pointing the same direction, minimizing steric hindrance. e.g. Figure S2b). For POM 3 with only 4 O_B_, it is computationally viable to consider all possible O_B_–H combinations with both dihedral angle directions, as only 250 calculations are required (Table S1). Herein we term these sets as O_B_^1φ^ limited to just one dihedral angle direction, and O_B_^2φ^ considering both dihedral directions. All three POMs were evaluated through 7 total degrees of reduction, considering both H atom reduction and alkylation (e.g., 5H + 2CH_3_ for POM 3).

The results reported herein focus on the thermodynamically preferred O_B_ H-binding sites, which are the same series of structures and resultant BDFE­(O–H) values previously reported.^41^ The structures are utilized subsequently for comparison purposes, statistical thermodynamic analysis, and bilinear modeling. In similar fashion as described above, select additional series of structures were resolved to provide more complete statistical thermodynamic representation of H-binding (particularly for POM 3), and we considered different counterion identities and/or coordinations (Figure S3). BDFE­(O–H) values were calculated from the DFT results by the following eqs 1–3

1 $$G_{DFT}=E_{Electronic}+G_{corr}$$

Where *G* stands for the Gibbs free energy, in kcal/mol. *G*_corr_ includes thermal internal energy, enthalpy, zero-point energy correction, and entropies from translation, rotation, vibration, per Gaussian Thermochemistry.^58^ The DFT BDFE­(O–H) and BDFE­(O–H)_avg_ may be calculated directly per eqs 2 and 3 below, using the lowest free energy configurations. Note that our experimental points of comparison are all BDFE­(O–H)_avg_ values, determined from pairs of clusters on the basis of 2H increments.^41^ Here *G* ^H^•^^ refers to the Gibbs free energy of the solvated hydrogen atom, and all components are solvated unless otherwise stated.

2 $$BDFE{\left(O-H\right)}_{x}^{DFT}=G_{\left(x-1\right)H}^{POM}-G_{xH}^{POM}+G^{H^{\cdot }}$$

3 $$BDFE{\left(O-H\right)}_{avg,x}^{DFT}=\left[G_{\left(x-2\right)H}^{POM}-G_{xH}^{POM}+2G^{H^{\cdot }}\right]÷2$$

here *x* = the number of H atom reductions of the cluster, and the *G*_*x*H_^POM^ are the *G*_DFT_ of the minimum-energy configurations for the respective degrees of H-binding. Equations 2 and 3 are the same as used in our previous work.^41^ Corresponding hydrogenation energies on either incremental or cumulative basis are calculated per eqs 4 and 5, respectively

4 $$\Delta G_{x,incremental}^{hydrogenation}=G_{xH}^{POM}-G_{\left(x-1\right)H}^{POM}-\frac{1}{2}G^{H_{2}\left(gas\right)}$$

5 $$\Delta G_{x,cumulative}^{hydrogenation}=G_{xH}^{POM}-G_{0H}^{POM}-\frac{x}{2}G^{H_{2}\left(gas\right)}$$

### 2.2 Ensemble Thermodynamics

Ensemble effects include *S* ^Config^ and Boltzmann averaging (BA) across the free energies of a population, *G* ^BA^. Each degree of H-binding to a POM affords a population of possible configurations. The ensemble *S* ^Config^ and *G* ^BA^ for these populations depend on the relative energies of their constituent POM configurations, which are calculated per eq 6, and their state probabilities, which are determined per Boltzmann statistics, eq 7

6 $$\Delta G_{i}^{rel}=G_{i}^{POM}-\min\left(G_{i}^{POM}\right)$$

7 $$p_{i}=\frac{e^{-\Delta G_{i}^{rel}/RT}}{\sum_{i}e^{-\Delta G_{i}^{rel}/RT}}$$

here “*i*” refers to the distinct configurations (states) for the same degree of H-binding and *p*_*i*_ is the normalized probability among all configurations with the same number of H-bindings, *R* is the gas constant, and *T* is the absolute temperature (298.15 K; room temperature). Δ*G*_*i*_^rel^ is the relative Gibbs free energy between specific configuration “*i*” and the lowest energy configuration [min­(*G*_*i*_^POM^)]. We assume the Ergodic hypothesis holds and that the individual state probabilities are equal to their ensemble fractional populations. These probabilities may then be used to determine the *S* ^Config^, via the Gibbs entropy equation (eq 8) and the Boltzmann average *G* ^BA^ per eq 9

8 $$S^{Config}=-R\sum_{i}p_{i}\;\ln\left(p_{i}\right)$$

9 $$G_{DFT}^{BA}=\sum_{i}p_{i}G_{DFT,i}^{POM}$$

Equation 9 is shown providing the Boltzmann average of the DFT values of the POM Gibbs free energies (*G*_DFT_^BA^) taken across the absolute DFT free energies (*G*_DFT,*i*_^POM^) directly. Prior works by our group^47^ and others^48–52^ note that to describe a complete ensemble, the contribution of *S* ^Config^ must be considered in addition to the DFT-calculated Gibbs free energies of individual structures. Thus, to account for the total free energy of a system, −TS^Config^ is incorporated as in eq 10

10 $$G_{Total}^{BA}=G_{DFT}^{BA}-TS^{Config}$$

Here *G*_Total_^BA^ is the Boltzmann average of the total Gibbs free energies of particles in the ensemble including the contribution of *S* ^Config^. We note that factoring the minimum energy structure of the ensemble, min. (*G*_DFT,*i*_^BA^), can be used to afford eq 11 below. This allows us to proceed and consider separately the effects from *S* ^Config^, and Δ*G* ^BA^; where Δ*G* ^BA^ is the difference between the ensemble Boltzmann average DFT free energy (*G*_DFT_^BA^) and the DFT minimum min­(*G*_DFT,*i*_^POM^), where the lowest energy DFT structures correspond to the same BDFE­(O–H) that were published in our previous work.^41^

11 $$G_{Total}^{BA}=\min\left(G_{DFT,i}^{POM}\right)+\Delta G^{BA}-TS^{Config}$$

This separation of terms is useful for subsequent thermodynamic analysis. Similarly, note that the Δ*G*_*i*_^rel^ and *p*_*i*_ per eqs 6 and 7 may be used to calculate Δ*G* ^BA^ directly

12 $$\Delta G^{BA}=\sum_{i}p_{i}\Delta G_{i}^{rel}$$

### 2.3 Statistical Thermodynamic Approximation of *S* ^Config^

Equations 8 & 12 may be used to calculate *S* ^Config^ and Δ*G* ^BA^ when the individual configurational probabilities (*p*_*i*_) or energies (*G*_*i*_^POM^) are known, i.e. when it is reasonable to resolve all relevant configurations via DFT. As noted above, this is achieved for POM 3 but is not practical for POMs 1 and 2 (Table S1). To determine the *S* ^Config^ and Δ*G* ^BA^ for POMs 1 and 2, we need to resolve representative distributions for their configurational free energies, for each degree of H-binding. Experience in prior work^41^ suggested small but non-negligible energetic differences exist between different configurations with the same type of O–H site binding, i.e. between H-bindings to different O_B_ sites. Therefore, we considered approximating the ensemble of different configurations via Boltzmann statistics, affording exponential decay distribution functions per the configurations’ relative energies (*vide infra* Figures 4, 6a, and S10–S12). A continuous mathematical expression for the probabilities of states analogous to eq 7 takes the form of^62^

13 $$p_{\left(y\right)}=\lambda e^{-y\lambda }$$

Here *y* is used to distinguish from the discrete states denoted with i above. λ is the decay parameter that describes how sharp or wide the distribution is. Smaller λ leads to wider distributions, and larger values afford shaper distributions. The continuous form of eq 13 suits substitution of *p*_(*y*)_ into eq 8 and integration across the continuous domain of *y*. This proceeds via eqs 14–16, and affords a tidy analytic expression for *S* ^Config^ per its distribution (eq 17)­

14 $$S^{Config}=-R\sum_{y}p_{\left(y\right)}\;\ln\left(p_{\left(y\right)}\right)$$

15 $$S^{Config}=-R\sum_{y}\lambda e^{-y\lambda }\ln\left(\lambda e^{-y\lambda }\right)$$

16 $$S^{Config}=-R\int_{0}^{\infty }\left[\left(\lambda e^{-y\lambda }\right)\times \ln\left(\lambda e^{-y\lambda }\right)\right]dy$$

The solution to this integral is

17 $$S^{Config}=R\left(1-\ln\;\lambda \right)$$

To further resolve these distributions, we need information about either *S* ^Config^, λ, or related quantities. As noted previously, for systems with large numbers of configurations such as POMs 1 and 2, it is impractical to solve them directly via DFT. However, the *S* ^Config^ and/or λ may instead be informed by the structural nature of H-binding to a POM, such as the possible numbers of configurations, and/or how those configurations affect the ensemble entropy.

From the above, one can deduce that as the number of relevant configurations in a distribution increases, the distribution grows wider, λ decreases, and *S* ^Config^ increases. Consistent with this behavior, it is well-known that for a system with isoenergetic (equiprobable) configurations, *S* ^Config^ can be approximated by^63^

18 $$S_{ST}^{Config}=R\;\ln\left(Q_{E}\right)$$

Where *Q*_E_ is the number of equiprobable configurations, i.e. the degeneracy. The subscript ST is used to denote that the value is approximated via statistical thermodynamics as opposed to DFT calculation. We note that the Boltzmann distributions describing our system (eqs 7 and 13) represent canonical ensembles and/or canonically equivalent multicomponent microcanonical ensembles (MC_Micro) with some number of nonisoenergetic configurations, while isoenergetic states would comprise microcanonical state counting;^64^ see Figure S4 for an illustration. However, we consider the limit in which the relevant number of configurations, Q, is restricted by the system temperature and nature of the distributions to a set of essentially degenerate, isoenergetic configurations. This is the case when some structural feature, e.g. presence vs lack of steric hindrance, creates an energetic difference between types of configurations, with system temperature sufficient to populate one type (i.e., with low energy) but not those with higher energy. Therein only one site type meaningfully characterizes the ensemble Gibbs free energy and changes to it (e.g., bridge site configurations without steric hindrance). Maintaining the number of particles, *N*, system volume, *V*, and energy, *E*, constant between MC_Micro and degenerate microcanonical ensembles requires that

19 $$E_{Micro}=E_{MC\text{\_}Micro}^{BA}=\sum_{i}^{MC\text{\_}Micro}\left[p_{i}\times E_{i}\right]$$

where “*i*” refers to the energetically diverse components of an MC_Micro ensemble, which has equivalent components to a canonical ensemble (see illustration Figure S4). Both the MC_Micro and canonical ensembles fit the configurational distributions we are considering herein (eqs 8 and 13). With this framework, equal *E*, *N*, and *V* across all three ensemble types implies that temperature, *T*, must also be equal, and that accordingly the ensembles will afford equivalent predictions for entropy, which is the case in the thermodynamic limit when accurately approximating the same macroscopic system.^63–68^ Thus, the microcanonical degeneracy in the effectively isoenergetic limit may be apportioned to a canonical distribution, as

20 $$\ln\left(Q_{E}\right)=-\sum_{i}p_{i}\;\ln\left(p_{i}\right)$$

Equivalent entropies follow mathematically, multiplying both sides by *R*. To justify the applicability of the thermodynamic limit, we note that we are working with experimental solutions having *N* on the order of 10^20^, that changes to the system size do not affect their intensive properties (i.e., extensive additivity holds), and that our systems are at equilibrium, have positive heat capacity, uniform phase, and are not near any phase transitions.^41,65–67,69^ Equivalent entropies enable us to consider eq 18 equal to eq 8, affording

21 $$R\left(1-\ln\;\lambda \right)=R\;\ln\left(Q_{E}\right)$$

Solving eq 21 affords

22 $$\lambda =\frac{e}{Q_{E}}$$

Here *e* is Euler’s number, approximately 2.71. Thus, if we determine a set of approximately isoenergetic configurations, *Q*_E_, we can describe the corresponding Boltzmann distribution and *S*_ST_^Config^. With the Boltzmann configurational distributions now characterized by their entropies, we can consider them further to determine Δ*G* ^BA^.

### 2.4 Statistical Thermodynamic Approximation of Δ*G* ^BA^

To determine Δ*G* ^BA^, we could proceed via analogous continuous approximation of eq 12 as we did with eq 8 for *S* ^Config^. However, considering equivalent domains for *p*_*y*_ and *p*_*i*_, one notes that the exponent of eq 13 (−*y*λ) is also equal to −Δ*G*_y_^rel^/*RT* per eq 7. In integrating the analogous continuous form across the full domain of *y* (eq 23), the *G*_*y*_^rel^ terms cancel, and the expression would just reflect the thermal energy in every case, with *RT* = 0.592 kcal/mol at the experimental conditions (298.15 K, 1 atm)

23 $$\Delta G^{BA}≲\int_{0}^{\infty }\left[\Delta G_{y}^{rel}\times \lambda e^{-y\lambda }\right]dy=RT$$

This may be satisfactory where *Q* is large, but not for systems where *Q* is small and the available states are further apart in energy such that higher energy states may not be visited. Thus, to determine the Δ*G* ^BA^, we instead consider a rediscretized distribution

24 $$p_{j}=\frac{e^{-j\lambda }}{\sum_{i}e^{-j\lambda }}$$

Workup of eq 24 is now aided per the above derivation that provides the λ values (eq 22). Here “*j*” is an index of states that runs from 0 to a satisfactorily large number, e.g. 10 × *Q*_E_, such that higher *j* would not significantly contribute to the ensemble population or average value calculations. The λ values parametrize the range over which *j* is relevant per eq 22. Additionally, using λ normalizes the functions consistently with the same *Q*_E_ state-counting. With probabilities calculated per eq 24, Δ*G*_ST_^BA^ may then be straightforwardly calculated per eq 12. With this practical framework in hand, we can now calculate ensemble *S* ^Config^ and Δ*G*_ST_^BA^ contributions to BDFE­(O–H) without excessive DFT requirements.

### 2.5 Bilinear Modeling of BDFE­(O–H)

Bilinear modeling was performed with the StatsModels^70^ Python module least-squares linear regression. Models with and without *S* ^Config^ and Δ*G* ^ΒΑ^ were considered. The bilinear interaction term was not found to be significant for these systems, and was thus precluded from the present models (coefficient set equal to zero). All final parameters were verified as significant (*p* < 0.01). All models were cross-validated with leave-one-out methods with respect to both individual BDFE­(O–H)_avg_ data points and POM sets of BDFE­(O–H)_avg_.

## 3 Results and Discussion

### 3.1 DFT Insights to POM BDFE­(O–H) Behavior

DFT investigation of 1H incremental H atom reductions, illustrated in Figure 2a, reveals values for the odd-numbered BDFE­(O–H), which were not attainable experimentally.^41^ These values help confirm the trend of decreasing BDFE­(O–H) with increasing degree of cluster H atom reduction, and highlight the overall linearity of these trends (Figure 2a). An analogous plot of experimental BDFE­(O–H)_avg_ is provided in Figure S5 for comparison. In Figure 2a, the BDFE­(O–H) trend clearly takes on similar slopes for POMs 1 and 2, suggesting that the BDFE­(O–H) trend is not substantially affected by the electronic effects of the ligand identity. This is consistent with the phenomena of thermodynamic compensation noted in our earlier work.^41^ Meanwhile, the sharper slope of POM 3 is made clear on a 1H basis (Figure 2a), further emphasizing another finding from our previous work, that the surface alkylation of POM 1 affording POM 3 has increased the magnitude of connection between the BDFE­(O–H) and the degree of cluster H atom reduction.^41^

To fully consider the linearity of the DFT BDFE­(O–H) trends, one notes in Figure 2a that the first BDFE­(O–H) for POMs 1 and 2 are lower than the second. This could reflect an electronic symmetry factor previously observed by the Matson and Miró groups for the singly reduced POMs.^40^ The relative symmetry of the non-hydrogenated cluster could stabilize it and thus reduce the first BDFE­(O–H); while symmetry breaking with the first protonation could slightly destabilize the 1H cluster. Together with an unpaired electron for the 1H cluster, this could favor some pairing with a second H atom reduction, slightly increasing its BDFE­(O–H). These findings suggest some impetus for these POMs to participate in PCET in 2H^+^/2e^–^ increments. This is consistent with previous findings on POM 1,^26^ the slope of the Nernstian open circuit potentials,^41^ and combination of experimental redox events into 2H^+^/2e^–^ PCET events.^36,71^ Taking the first two metal center reductions on their average, the remaining BDFE­(O–H) points and trends suggest little deviation from linearity.

Figure 2b expands upon our previous work^41^ by underscoring that while sufficient for corroboration, compared to experimental findings the raw DFT calculations captured only part of the BDFE­(O–H) trend (45% on average) and exhibit an overall root mean squared error (RMSE) of 2.35 kcal/mol. Additionally, offsets vs parity are apparent on a per-cluster basis. This motivates our current work, and we demonstrate how ensemble effects (commonly neglected in DFT calculations) can give rise to deviations between experiments and theory.

First expanding the scope of DFT investigation from our prior collaborative work,^41^ we considered additional H-binding configurations (Figure S2a and Table S1), counterion identities and positions (Figure S3). An overview of the different oxygen site energetics (lowest energy structures for the specific binding site types) is provided in Figure 3, displaying the cumulative energies of hydrogenation for incremental O–H binding positions. The different possible H-binding site types (Figure 1a, blue circles) correspond to different incremental energies of hydrogenation (Figure 3)­, which mostly remain consistent throughout full occupation of the O_B_. Consistent with our previous findings^41^ Figures 3 and S6 for POMs 2 and 3 illustrate that O_B_ sites are thermodynamically preferred, and that cluster hydrogenation is exergonic through full occupation of the available O_B_ sites (6 for POMs 1 and 2; 4 for POM 3), corresponding to 1-electron reduction of all initial V^V^ atoms. Additionally, Figures 3 and S6 show that the incremental hydrogenation energies and their linear trends are substantially constant for cluster reductions up to 6 V^IV^.

**Figure 3:**
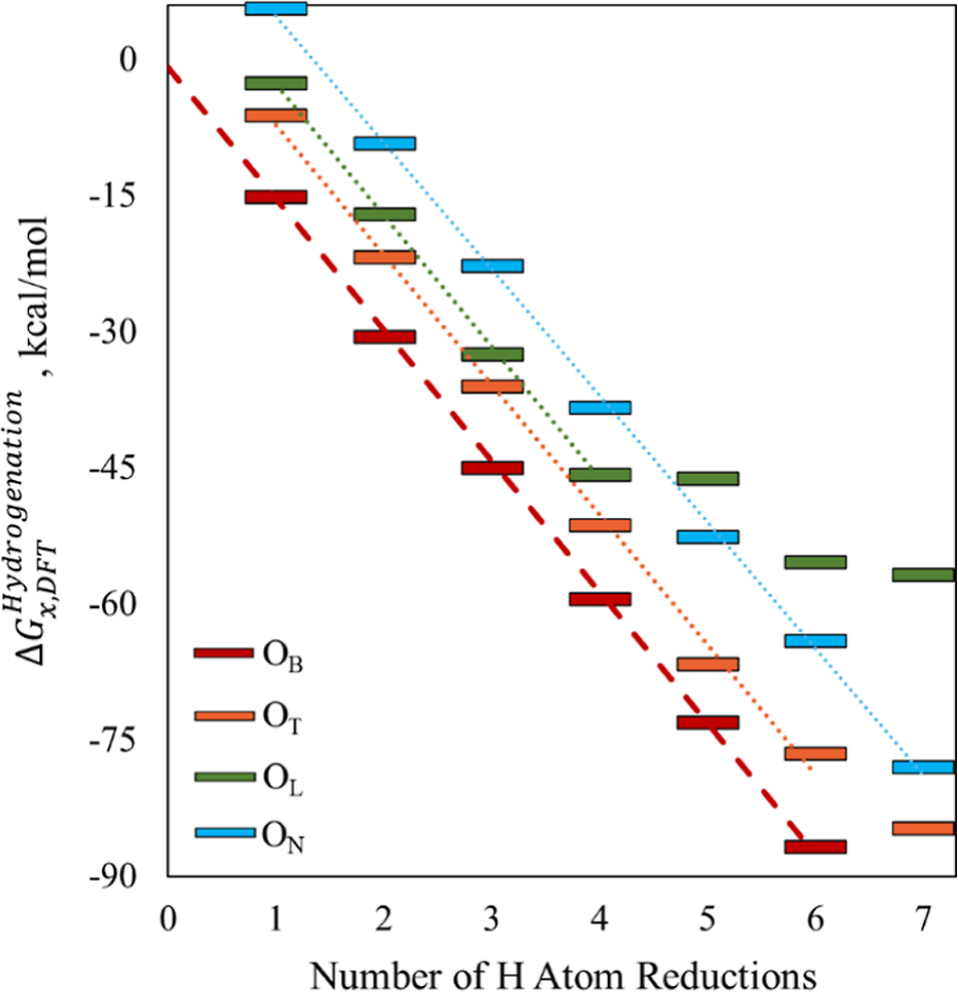
Cumulative Gibbs free energies of hydrogenation with different incremental H-binding locations on POM 1, relative to the 0H cluster, per eq 5. The configurations for each successive H-binding are based upon the preceding lowest energy configuration.

These results are important for understanding prospective PCET energetics of bulk vanadium oxides, as the nanocluster vanadium-oxygen bond types are very similar to those found on bulk system surfaces.^72^ Both molecular and bulk system surfaces feature O_B_ and O_T_ sites (Figures 1 and S7) and may be analogously ligand-modified and/or hydrogenated. The incremental hydrogenation energies correlate to the BDFE­(O–H) via eqs 2–5, which change in reference from 1/2H_2_(*g*) hydrogenation to solvated H atom reductions. O_T_ sites are consistently found to be second-most thermodynamically favored for H-binding, but at least 6 kcal/mol less favorable than O_B_ sites if O_B_ are available. An important consequence thereof is that the relative ensemble populations of O_T_–H and higher energy states at ambient conditions is much lower than O_B_ configurations, on the order of <50 ppm per eq 7. Similarly, the incremental hydrogenation energies beyond 6 V^IV^ in the clusters correspond to non-O_B_ sites and are much smaller, by at least 10 kcal/mol. Using different counterions or different counterion positions (Figure S3) did not substantially affect the hydrogenation trends. Considering variable counterion positions within a series was found to have only minor effect on incremental energies, <1 kcal/mol (Figure S8), attributed to minor differences in cluster-counterion interaction and O—H-counterion steric hindrance. None of the variations considered significantly improved experimental BDFE­(O–H)_avg_ trend-capture.

Inspection of the individual DFT *E*_Electronic_ and *G*_corr_ terms reveals that the vast majority of the BDFE­(O–H) and its trends with H atom addition arise from differences in *E*_Electronic_ (Figure S9). These *G*_corr_ values utilize standard harmonic analysis.^58,59^ Anharmonic vibrational analyzes were also considered to refine *G*_corr_, but for these POM systems, each anharmonic calculation required inordinately large computational resources and considering them across ensemble sets was found to be prohibitive. Moreover, anharmonic calculations would be expected to slightly lower *G*_corr_ values systemically on an absolute basis (e.g., about 5%^73^), but literature suggests that the anharmonic analysis results may be comparable to uniform scaling of harmonic values,^74^ and thus would not excessively impact the *G*_corr_ relative energetics. The corresponding BDFE­(O–H) are incremental values, where systemic absolute energy changes would be expected to largely cancel-out. While the magnitude of anharmonic corrections could be significant if cancellation is incomplete for some structures, such cases could also be less accurate.^74^ We find that with harmonic treatment, structures differing only in single successive H-binding have consistent *G*_Corr_ increments of about 6 kcal/mol. Noting that herein we consider multiple configurations for each degree of H-binding, we would not expect significant differences to arise in the increments between sets due to near-systemic potential 5% refinements.^73^ Therefore, improvement to the BDFE­(O–H) and their trend accuracy is not expected, and we did not further pursue anharmonic calculations in the present work. Thus, we maintain the consistent counterions, methods, and structures as used in our previous work^41^ (Figure 1) and focus on H–O_B_ configurations for cluster reductions up to 6 V^IV^.

### 3.2 Ensemble Features, *S* ^Config^, and Δ*G* ^BA^

As discussed in the Computational Methods section, the DFT values presented in Figure 2a,b are derived from the lowest free energy configurations for each degree of H atom reduction (Figures 3 and S6). With the free energy minima as basis, the relative free energies and ensemble probabilities are then calculated for each configuration investigated, per eqs 6 and 7. By using DFT we can resolve the full ensemble populations for each relevant H-binding configuration with POM 3 (Figure 4). For POMs 1 and 2 we are limited in practice to the set of O_B_^1φ^; we provide normalized ensemble probabilities for these in Figures S10–S12. The various O_B_–H configurations have significant thermal occupation probabilities because their Δ*G*_i_^rel^ are small, generally within 2 kcal/mol of the minima. This energetic accessibility is what gives rise to the ensemble populations and their effects on the final measured properties. In the ensemble distributions (Figure 4 and S10–S12), each degree of H atom reduction displays a decaying probability distribution for its different configurations per their relative energies, consistent with Boltzmann populational decay features for distinct states. For POMs 1 and 2 (Figures S10 and S11) as expected per “6 O_B_ choose #H” combinatorics, the number of possible H-binding configurations increases from #H = 1 to a maximum number of combinations for #H = 3, and then decreases to just 1 possible combination for #H = 6. Figures 4 and S12 illustrate similar behavior for POM 3, and one may note the difference in POM 3 ensemble resolution between using the O_B_^2φ^ set (Figure 4) and limited O_B_^1φ^ set (Figure S12). These decaying probability distributions are consistent with our employed canonical distributions (see Computational Methods, eq 13).

With changes in the number of configurations for each degree of H atom reduction, we expect changing ensemble effects from *S* ^Config^ and concomitant Δ*G* ^BA^ to emerge and influence the system average H-binding energies. Consideration of both the DFT minimum energy configuration and ensemble contributions to the BDFE­(O–H) proceeds via eq 25, which is derived from inserting *G*_Total_^BA^ of eqs 11 into eq 2 twice, once for both *x* and (*x* – 1) successive degrees of H-binding

25 $$BDFE{\left(O-H\right)}_{x}^{Ensemble}=BDFE{\left(O-H\right)}_{x}^{DFT}+T\Delta S_{x}^{Config}-\Delta \left(\Delta G_{x}^{BA}\right)$$

Here Δ*S*_*x*_^Config^ = *S*_*x*H_^Config^ – *S*_(*x*–1)H_^Config^ and reflects the energetic impact of accounting for the configurational entropies, and Δ (Δ*G*_*x*_^BA^ = Δ*G*_*x*H_^BA^ – Δ*G*_(*x*–1)H_^BA^) represents the impact of ensemble population Boltzmann average energetics vs DFT minimum free energies. Based on the POM structures (Figure 1), we considered different possible combinations of the O–H configurations as potential *Q*_E_, including

26 $$Q\left(O_{B}^{1\varphi }\right)=\left(\begin{array}{l} \#\;of\;O_{B}\\ \#\;of\;H \end{array}\right)$$

27 $$Q\left(O_{B}^{2\varphi }\right)=\left(\begin{array}{l} \#\;of\;O_{B}\\ \#\;of\;H \end{array}\right)\times 2^{\left(\#\;of\;H\right)}$$

28 $$Q_{NS}=Q\left(O_{B}^{2\varphi }\right)-\#\;of\;Steric\;Configs$$

As described in the Computational Methods section, the *Q*(*O*_B_^1φ^) and *Q*(*O*_B_^2φ^) sets consider: (1φ) only 1 dihedral O–H direction, or (2φ) both dihedral O–H directions, respectively. “# of H” stands for the number of H-bindings to the cluster. “# of O_B_” stands for the number of cluster O_B_ that are available for H-binding (i.e., that are only bonded to two V atoms). *Q*_NS_ stands for the number of O_B_^2φ^ configurations that have no steric hindrance between adjacent O–H groups. For each degree of H-binding, the set of Q_NS_ can be considered as self- and neighbor-avoiding for successive H-binding, for which a concise analytical expression is not known.^75^ Thus, the number of energetically relevant (*Q*_NS_) and/or sterically hindered configurations (# of Steric Configs) were enumerated via analysis of combinatorial graphical representations (e.g., Figures S13 and S14) (Table S1).

**Figure 4:**
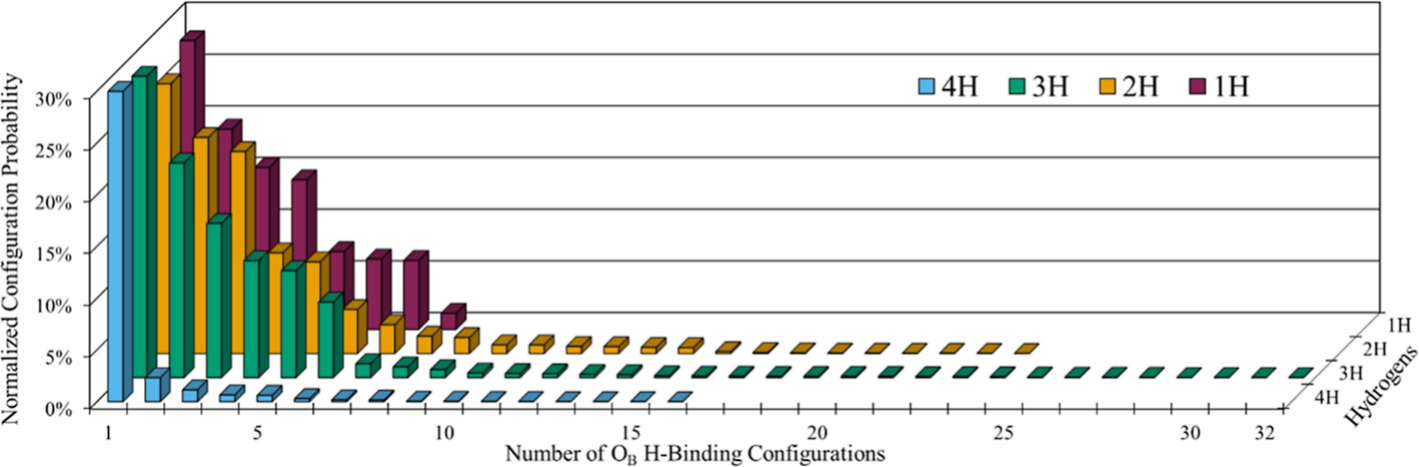
Normalized POM 3 ensemble distributions per DFT evaluation of the complete configurational space, the O_B_^2φ^ set, with two possible hydrogen-binding configurations per O_B_ atom (250 DFT calculations, Table S1). Probabilities were determined via Boltzmann statistics (eq 7). Each value displayed represents a distinct possible combination of H-binding to different O_B_ atoms. The #H order is shown from 4H to 1H (foreground to background) and the *y*-axis is limited to 30%, both for clarity. The first 4H configuration comprises 94% of its normalized distribution, comparable to the sole configuration of POM 3 with 0H. Ensemble distributions for POMs 1, 2, and 3 on an O_B_^1φ^ basis are provided in Figures S10–S12.

In succession we explored the impact of including *S* ^Config^, specifically the *T*Δ*S*_ST_^Config^ term of eq 25, using the combinatoric number of states per *Q* (O_B_^1φ^) (eq 26), *Q* (O_B_^2φ^) (eq 27), and *Q*_NS_ (eq 28). Their impacts on BDFE­(O–H) are illustrated in Figure 5, relative to zero for the DFT-only results without *S* ^Config^ effects. For POM 3, DFT resolution of all possible configurational combinations is practical (Table S1), and we can compare the value of *S* ^Config^ per DFT calculations and eq 8 vs our statistical thermodynamic construct (eq 18) utilizing either *Q* (O_B_^1φ^), *Q* (O_B_^2φ^), or *Q*_NS_ (Figure 5a; light blue triangles, purple hexagons, and orange Xs, respectively). For POMs 1 and 2 where full DFT resolution is not practical (Table S1), we instead focus on how incorporating *T*Δ*S*_ST_^Config^ into BDFE­(O–H) helps close the trend gap between DFT and experimental. We visualize this in Figure 5b with the average trend gap illustrated as a black diagonal, representing how much *T*Δ*S*_ST_^Config^ would need to impact the BDFE­(O–H) in order to close the trend-capture gap of Figure 2b. Utilizing either *Q* (O_B_^1φ^) or *Q* (O_B_^2φ^) was found to improve trend-capture (Figure 5), with slightly higher magnitude impacts for *Q* (O_B_^2φ^). This suggests that the number of relevant sites does depend on the O_B_ atom combinatorics, and on 2 sites per O_B_ atom. However, both *Q* (O_B_^1φ^) and *Q* (O_B_^2φ^) still underestimated the *T*Δ*S*_ST_^Config^ impact calculated for POM 3 (Figure 5a), and leave about 40% of the DFT-experimental trend gap unaddressed (Figure 5b).

With higher degrees of H-binding, an increasing number of O_B_–H configurations become energetically disfavored by steric interactions, and the number of energetically accessible configurations separates from that which would be dictated by 2^N^ combinatorics (eq 28 vs [Disp-formula eq27]), pointing to larger *S*_ST_^Config^ differences between degrees of H-binding with *Q*_NS_. Considering the impact of *T*Δ*S*_ST_^Config^ per *Q*_NS_ aligns almost perfectly with both determination via DFT and eq 8 for POM 3 (Figure 5a), and with closure of the DFT-experimental trend gap for POMs 1 and 2 (Figure 5b). We note that some offsetting errors may participate in the apparent degree of closing trend gaps. For example, Boltzmann average effects are not yet included for POMs 1 and 2, and the individual DFT points of POM 3 vary about its trend. However, the *Q*_NS_ set (eq 28) reflects the POM structural features, improves upon less rigorous relevant configuration enumeration (eqs 26 and 27), and moreover, such potential offsetting errors would not trivialize its significant improvement in trend-capture compared to a single-structure DFT baseline. We further assessed alignment of the *Q*_NS_ construct compared to the available O_B_^1φ^ sets of DFT data, and found consistent fit between *Q*_NS_ predictions, the POM 3 set, and consistent relative alignment to the O_B_^1φ^ sets for all POMs as one may expect per the differences in completeness between O_B_^1φ^ and O_B_^2φ^ sets (Figures S15–S17). With *Q*_NS_, in Figure 5 one notes that all three POMs have the largest magnitude *S* ^Config^ effects at either end of the H-binding series. This follows mathematically from eqs 18, 25, and 28. The similar magnitudes toward either end are consistent with mirroring combinatoric effects (eq 26), although we note that *Q*_NS_ is not perfectly symmetrical about the series middle due to increased possible combinations being incompletely offset by steric factors (Table S1, column *Q*_NS_); e.g. POM 1 with 1H has *Q*_NS_ = 12, while POM 1 with 5H a.k.a. one “hole” has *Q*_NS_ = 36 (eq 28). Thus, considering *Q*_NS_ = *Q*_E_ of our Computational Methods provides a structural ensemble-informed basis and satisfactory fit to help close the gap between DFT and experimental BDFE­(O–H) trends (Figure 5).

**Figure 5:**
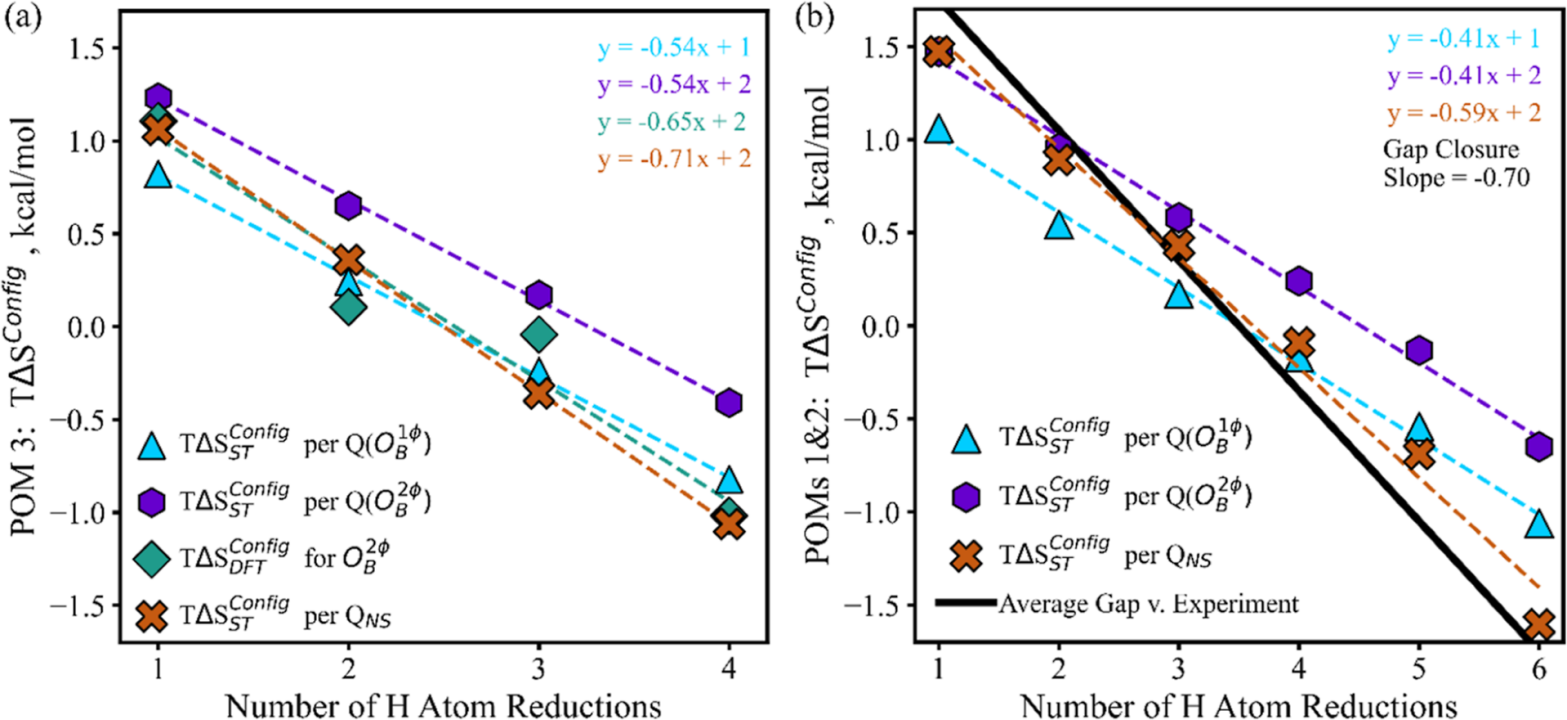
Impact of different determinations of *S* ^Config^ (a) in aligning with the results of full DFT resolution of the configurational space for POM 3 (Table S1, 250 calculations), and (b) in closing the DFT-experimental average trend gap for POMs 1 and 2. Note the DFT-alone baseline is equal to 0 on the *y*-axis. In (a) the gap between DFT and experimental trends for POM 3 is 0.64 kcal/mol of BDFE­(O–H) per H reduction; line omitted for clarity. The trend gaps are considered as the average difference between the DFT and experimental slopes of the BDFE­(O–H) vs #H atom reduction trends for POMs 1 and 2, centered at the H atom reduction series midpoints. Thus, the *y*-axis quantifies the differences for the trend gap vs experiments and of the various *Q* series vs the DFT-alone trends.

With *Q*_NS_ identified as *Q*_E_ for our POM systems, we proceeded to calculate Δ*G* ^BA^ as described in our Computational Methods. This approach was first tested using *Q*_NS_ with eqs 22 and 24 to calculate Δ*G*_ST_^BA^ and compare against the computationally tractable POM 3 full DFT results (Figure S16). An illustration of the distribution from our statistical treatment in comparison to the DFT afforded values is provided for the 1H POM 3 in Figure 6a. Analogous distributions were generated for the other degrees of POM 3 H-atom reduction, and the calculated Δ*G*_ST_^BA^ agree excellently with the Δ*G*_DFT_^BA^ (Figure 6b). We proceed to calculate Δ*G*_ST_^BA^ by the same method for POMs 1 and 2 with *Q*_NS_. We find that it captures at least the partial Δ*G*_DFT_^BA^(O_B_^1φ^) that we were able to calculate via DFT for these POMs (Figure S15b), and attains similar ratio of capture as the POM 3 values for Δ*G*_DFT_^BA^(O_B_^2φ^) vs Δ*G*_DFT_^BA^(O_B_^1φ^) (Figure S16a).

The impact of considering Δ*G*_DFT_^BA^ on the BDFE­(O–H) per eq 25 is illustrated for POMs 1 and 2 in Figure S15b and for POM 3 in Figure S16a. Consideration of Δ*G*_DFT_^BA^ primarily affects the first and final BDFE­(O–H), as may be expected for the corresponding differences between single relevant molecular configurations (for empty and full O_B_ occupations) vs ensembles of partially H-bound O_B_. Increasing the consideration of ensemble effects from partial O_B_^1φ^ to full O_B_^2φ^ increases the magnitude of impact for both *S* ^Config^ and Δ*G* ^BA^ (Figures 5, S15, and S16a). This is observable in the slopes of each of the *S* ^Config^ and Δ*G* ^BA^ trends, as well as in the total calculated ensemble BDFE­(O–H) values (eq 25) (Figures S16b and S17). The statistical thermodynamic approach (orange X data points) aligns excellently with the full ensemble DFT data for POM 3 (Figure S16b), and is consistent in relative alignment to the available POM 1 and 2 O_B_^1φ^ data (Figure S15b). With *T*Δ*S*_ST_^Config^ and Δ*G*_ST_^BA^ determined, we may now examine their influence on the parity between theoretical and experimental results.

**Figure 6:**
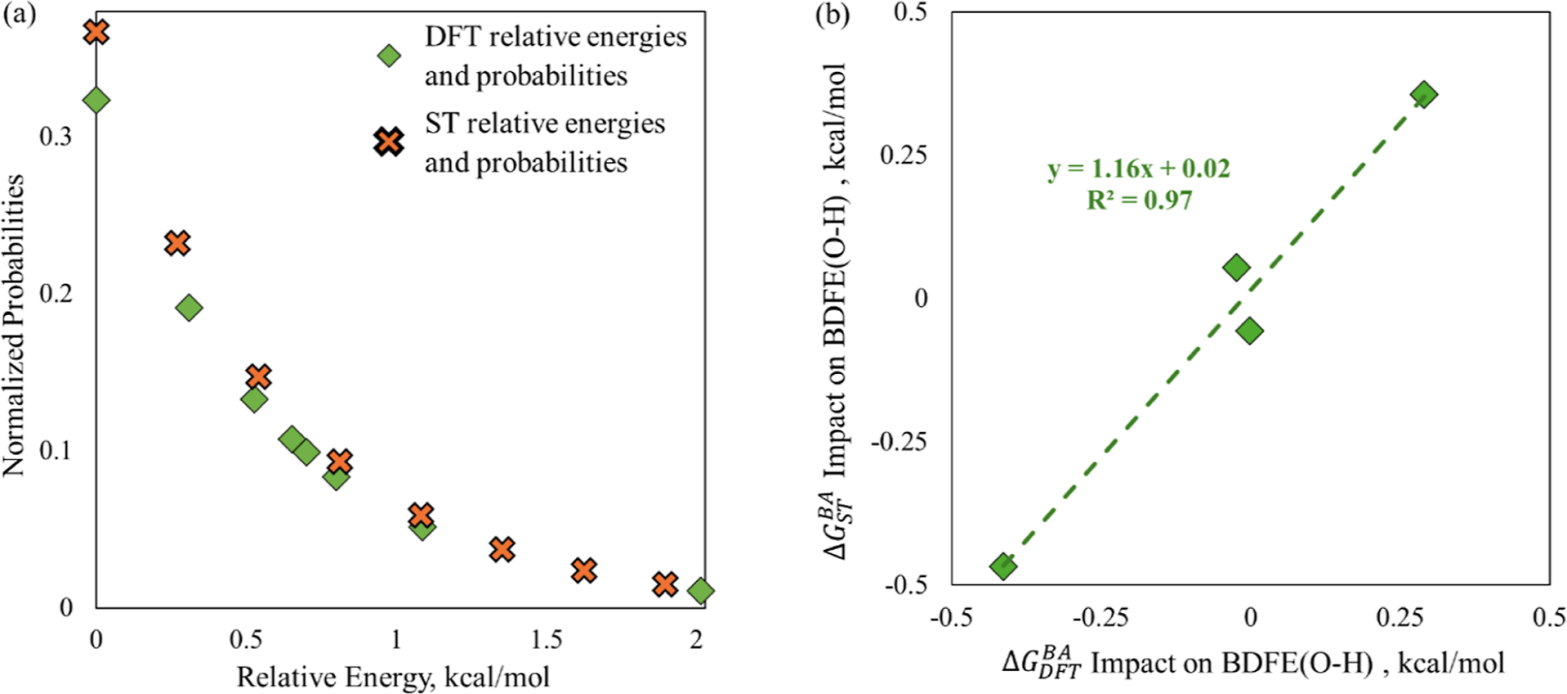
Fit of the statistical thermodynamic calculation of relative energies and probabilities vs POM 3 O_B_^2φ^ DFT data for (a) all O_B_^2φ^ 1H configurations, and (b) all POM 3 Δ*G*_ST_^BA^ vs Δ*G*_DFT_^BA^.

### 3.3 Experimental Parity Improvements from Ensemble Effects, Bilinear Modeling

The result of including *T*Δ*S*_ST_^Config^ on the parity of calculated BDFE­(O–H)_avg_ values to experimental values is illustrated in Figure 7a. This inclusion approximately doubles the degree of average trend-capture (from 45% to 91%). In the evolution of the per-cluster slopes from Figures 2b to 7a, one observes for all three POMs that the magnitude of the *T*Δ*S*_ST_^Config^ impact on the BDFE­(O–H) trend is approximately equal to the magnitude of the BDFE­(O–H) trend of the initial DFT determined free energy values. This suggests that consideration of ensemble effects and specifically *S* ^Config^ can improve the BDFE­(O–H) calculation for hydrogenated metal oxides. We therefore revisit the ceria nanocrystals investigated by Agarwal, Kim, and Mayer.^42^ Therein we considered similar possible H-binding sites per each surface O, and from evaluating the *S*_ST_^Config^ magnitude of the first H-binding, we find that *S* ^Config^ could account for between 1/5 and 1/2 of their BDFE­(O–H) trends with cerium reduction (Table S2, Figure S18). This would help explain a corresponding portion of the behavior in alignment with the Frumkin isotherms Agarwal et al. determined.^42^

The inclusion of Δ­(Δ*G*_ST_^BA^) somewhat offsets the trend impact of including *T*Δ*S*_ST_^Config^, by about one-fifth on average, but tightens the data around each per-cluster trend, and reduces the overall RMSE of the calculated BDFE­(O–H) ensemble values vs parity with experiments (Figure 7b vs 7a). Additionally, in Figure 7b we see that the per-cluster trends now do not cross, that the trends maintain approximately equal differences between the entireties of their trendlines, and that the magnitudes of the per-cluster trend slopes also align with the magnitudes of their offsets vs parity. This clear per-cluster behavior in both slope and offset vs parity suggests another factor varies on a per-cluster basis and correlates with these offsets.

**Figure 7:**
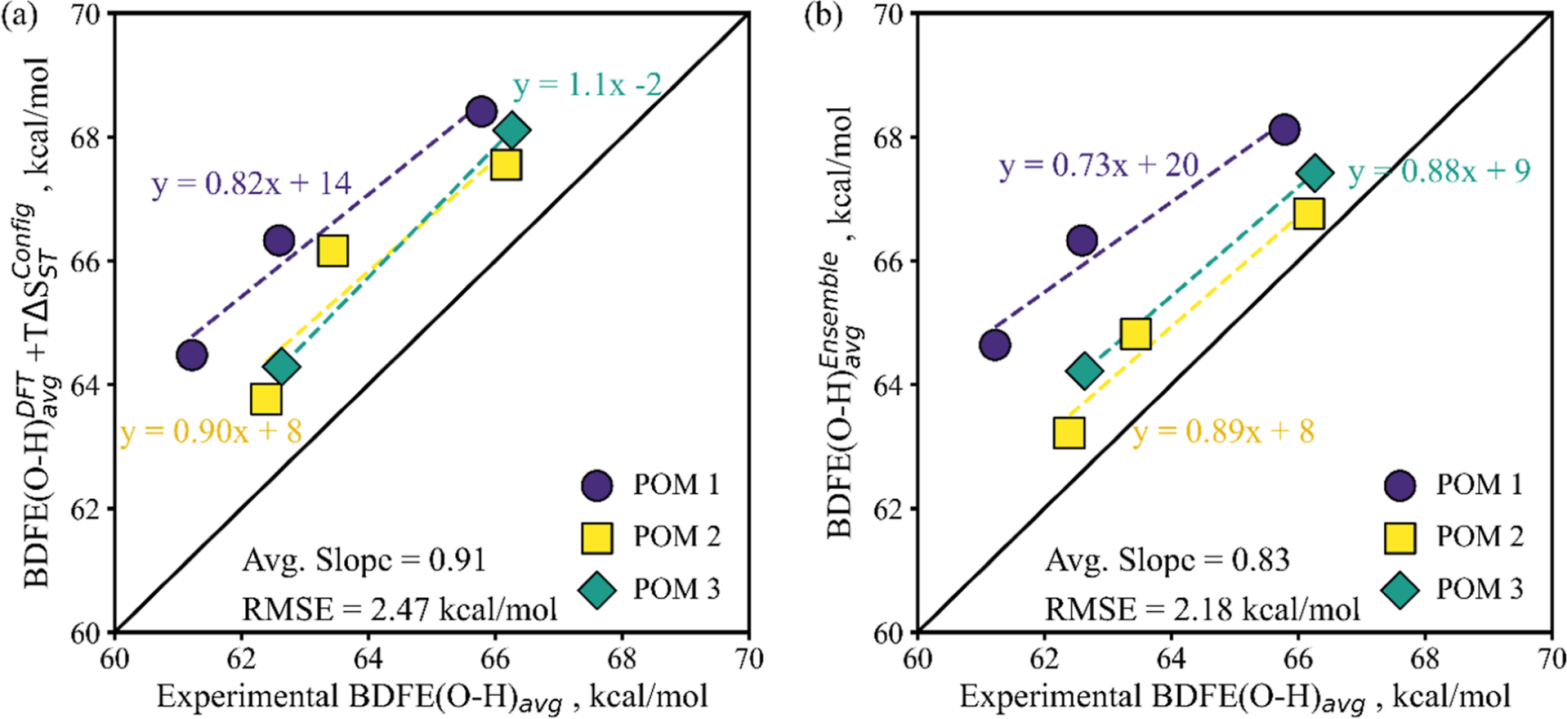
*S*_ST_^Config^ and Δ*G*_ST_^BA^ effects on DFT BDFE­(O–H)_avg_: (a) DFT BDFE­(O–H)_avg_ with *S*_ST_^Config^ per eq 18 and *Q*_NS_, not including Δ*G*_ST_^BA^, and (b) DFT BDFE­(O–H)_avg_ including *S*_ST_^Config^ as well as Δ*G*_ST_^BA^ per eq 25. The POM data points represent the same numbers of hydrogen atom reductions in the same order as in Figure 2b, labels omitted for clarity.

As discussed in our introduction, we were aware of the different ligands’ per-cluster effects on their molecular electronics^40^ and that the experimental BDFE­(O–H)_avg_ found in the complex electrochemical solutions correlated with average O_B_ charge transfer.^41^ Work by Hait and Head-Gordon suggested that electron affinities may be used as a modifying parameter, owing to their relation to electron delocalization.^76^ This motivated us to investigate potential correlations between the per-cluster offsets and appropriate modifying parameters connected to the local electronic environments. The average O_B_ charges of the clusters at the same oxidation state (with 6 V^V^ centers) were found to correlate excellently with the per-cluster offsets vs experimental parity (Figure 7b). This correlation follows logically as it captures the changes to the electronic environment as a result of differing ligation, separate from V reduction. In addition, it aligns with the observed shifts in the O_B_ charges for reduced POM 3 clusters in the trend vs BDFE­(O–H)_avg_ in our prior work.^41^ As noted previously, some differences between the complex experimental system and implicit solvation DFT electronic environments are reasonable.^41^ Because the 6 V^V^ average O_B_ charges provide descriptors of the ligand chemical effects on the local electronic environment, in correlation with the DFT offsets vs experimental parity, they may be used in a bilinear model to account for the corresponding differences in local electronic environments, and thus reconcile the DFT BDFE­(O–H)_avg_ vs experimental values. The resultant bilinear model follows in the form of eq 29. It is entirely DFT/ST based, with minimal computational cost and high accuracy (Figure 8a).

29 $$BDFE{\left(O-H\right)}^{model}=1.19\times BDFE{\left(O-H\right)}^{Ensemble}-286\;\frac{kcal/mol}{Charge}\times \left[{O_{B}}_{6V^{V}POM}^{Avg\text{\_}Charge}+0.451\right]$$

Equation 29 includes a 1.19 parametrization of the BDFE­(O–H)^Ensemble^ calculated per eq 25, and −286 $\frac{kcal/mol}{Charge}$ times the per-cluster 6 V^V^ average O_B_ charge ( ${O_{B}}_{6V^{V}POM}^{Avg\text{\_}Charge}$ ) relative to the model intercept for the 6 V^V^ O_B_ charge at −0.451. The 6 V^V^ average O_B_ charges for POMs 1, 2, and 3 are −0.395, −0.404, and −0.401, respectively (NBO charges, as discussed in Computational Methods). Bilinear model BDFE­(O–H)^Model^ values on a 2H average basis are presented vs the respective experimental BDFE­(O–H)_avg_ in Figure 8a. The BDFE term almost exactly closes the remaining trend parity gap between the calculated BDFE­(O–H)^Ensemble^ and the experimental values for this complex environment (Figures 8a vs [Fig fig7]b). The O_B_ charge term is limited to adjusting the absolute BDFE­(O–H) values on a per-cluster basis and does not change the BDFE­(O–H) trend slopes vs parity. This is as-desired for a correction based on the local electronic environment: the model terms are limited to specific BDFE­(O–H) and per-cluster O_B_ charge components, avoiding any other tuning that could result in overfitting. We further evaluated and cross-validated bilinear models (using ${O_{B}}_{6V^{V}POM}^{Avg\text{\_}Charge}$ ) with (i) unmodified DFT BDFE­(O–H)_avg_, (ii) DFT BDFE­(O–H)_avg_ plus the impact of *S*_ST_^Config^, and (iii) BDFE­(O–H)_avg_ plus the impact of both *S*_ST_^Config^ and Δ*G*_ST_^BA^ (as in eq 25 for BDFE­(O–H)_avg_^Ensemble^). Bilinear modeling achieved excellent parity in all cases (Figures 8a and S19). We performed cross-validation with leave-one-out-data point (Figures S20 and S22a) and leave-out-one-POM (Figures S21 and S22b). The bilinear model (eq 29) utilizing the BDFE­(O–H)^Ensemble^ (eq 25) performed the best in its overall fit (Figure 8a), leave-out-one-data point cross-validation (Figure S22a), and leave-out-one-POM cross-validation (Figure S22b); although all models realized improvements in RMSE to <1.5 kcal/mol or better. Thus, relative to DFT alone (Figure 2b), the ensemble accounting first (eq 25), and bilinear modeling second (eq 29), have reduced the RMSE by 85% and improved the BDFE­(O–H) trend-capture from 45% to 96% (Figure 8a).

**Figure 8:**
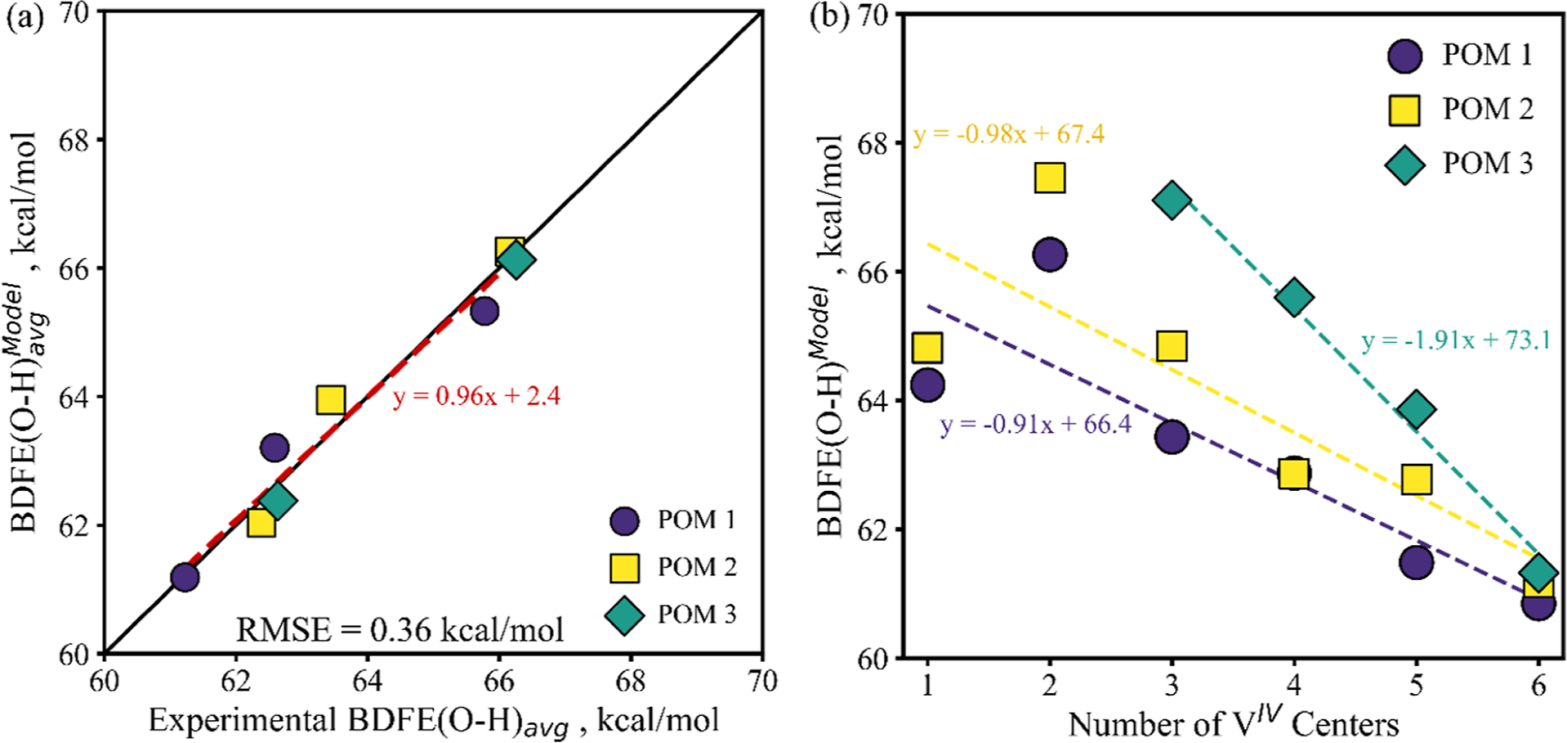
Bilinear model (a) BDFE­(O–H)_avg_^model^ parity vs experimental BDFE­(O–H)_avg_, and (b) BDFE­(O–H)^Model^ values vs the number of V^IV^ centers. BDFE­(O–H)^Model^ values are calculated per eq 29, including *S*_ST_^Config^ and Δ*G*_ST_^BA^ per eq 25. (b) May be directly compared with Figure S5 experimental values and trends.

We note that our present model is finely tuned to accuracy per the available data of the three subject POM systems. However, the same approach should be extensible to other systems (results not shown here). POMs and their BDFE­(O–H) are an active area of investigation for our group (e.g., ref 36). We acknowledge that using the same presently precise model for other systems may require refitting of the bilinear model coefficients for high accuracy. Nevertheless, as noted above, our previous work^41^ identified a strong correlation between the BDFE­(O–H)_avg_ and O_B_ charges for the subject POMs. Similar connections to the local electronic environments have been found for bulk WO_3_, TiO_2_ and other systems by our group^36,77^ and others.^78^ The relative magnitudes of ensemble effects may change from one system to another, but their manifestation is fundamental from statistical mechanics^63,64^ and their importance has been noted in various systems.^46–50^ Overall, the prevalence of similar phenomena suggests broad potential applicability of similar approaches.

Applying the bilinear model to the 1H increment BDFE­(O–H) of Figure 2a produces Figure 8b, with improved fits vs both experimental trends and absolute BDFE­(O–H)_avg_ values (compare with Figure S5). With more precise experimentally correlated 1H increment data, we can now more closely examine phenomena with smaller BDFE­(O–H) changes. Notably, the Figure 8b trends of BDFE­(O–H) vs the number of H atom reductions are very linear. This suggests that so long as similar metal centers are reduced and O sites protonated, the BDFE­(O–H) trend may be approximated linearly. This may enable great reduction of computational requirements to resolve BDFE­(O–H) for subsequent clusters. If the trend slope is determined, then one only needs to apply DFT to calculate the energies of the series end points. An example of this is included in the Supporting Information (Figure S23); wherein the number of required DFT calculations for the same three subject POMs is reduced from at least 4260 to consider all configurations, to just 84, a reduction of 98%.

## 4 Conclusion

We present experimentally consistent DFT-based statistical thermodynamic models of BDFE­(O–H) on polyoxometalates, including *S* ^Config^, ensemble free energy averages, and local electronic effects. Importantly, we reveal that an array of energetically accessible O–H binding configurations give rise to ensemble effects even at room temperature (experimental conditions), and that accounting for these effects describes accurately the POM cluster BDFE­(O–H) trends with H reduction. Considering the ensemble effects together with bilinear adjustment for the local electronic environment achieves great accuracy vs experimental values, with RMSE < 0.4 kcal/mol.

In determining cluster ensemble effects, we found that the set of energetically accessible configurations essentially excludes configurations with steric hindrance between adjacent O–H bindings. The impact of *S* ^Config^ helps explain a majority of previously obscure differences between higher magnitude experimental BDFE­(O–H) trends vs lower predictions from individual theories such as the Nernst equation. Bilinear reconciliation per differing electronic environments of the clusters, as depicted by their O_B_ charges at the same oxidation state, systematically adjust the cluster BDFE­(O–H) series on a per-cluster basis, essentially closing previous offsets between theory and experiment that ranged from 1 to 4 kcal/mol in magnitude. We consider that our model is carefully optimized to the present systems. However, we noted potential relevance to other systems, and because our model’s features are rooted in physics, we expect its principles to be generally applicable. Additionally, we demonstrated that the BDFE­(O–H) of intermediate degrees of H-binding may be estimated from structurally informed statistical thermodynamics and DFT resolution of just the first and last BDFE­(O–H) of a linear series.

The methodology applied herein offers a powerful tool to rapidly explore novel POM system thermodynamics relevant to PCET and reduce computational requirements for their accurate estimation. These findings can help advance research in fields where PCET is a central process, including catalysis of hydrogen evolution, selective hydrogenations, and other important redox and catalytic systems.

## Supplementary Material



## Notes

Relative energies of different states, O–H binding dihedral orientations, POM structures, ensemble distributions, modeling of different impacts on BDFE­(O–H), bilinear modeling, leave-one-out analyzes and cross-validated models (PDF)

## References

[1] Huynh M. H. V., Meyer T. J. (2007). Proton-coupled electron transfer. Chem. Rev..

[2] Nocera D. G. (2022). Proton-Coupled
Electron Transfer: The Engine of Energy Conversion and Storage. J. Am. Chem. Soc..

[3] Korotcenkov, G. Metal Oxide Series; Elsevier, 2017–2023.

[4] Gumerova N.
I., Rompel A. (2018). Synthesis,
structures and applications of electron-rich
polyoxometalates. Nat. Rev. Chem..

[5] Horn M. R. (2021). Polyoxometalates (POMs): from electroactive clusters to energy materials. Energy Environ. Sci..

[6] Proe K. R., Schreiber E., Matson E. M. (2023). Proton-Coupled Electron Transfer
at the Surface of Polyoxovanadate-Alkoxide Clusters. Acc. Chem. Res..

[7] Hammes-Schiffer S. (2015). Proton-Coupled
Electron Transfer: Moving Together and Charging Forward. J. Am. Chem. Soc..

[8] Klein J. E. M. N., Knizia G. (2018). cPCET versus HAT: A Direct Theoretical Method for Distinguishing
X-H Bond-Activation Mechanisms. Angew. Chem.
Int. Ed..

[9] Ullmann, F. ; Gerhartz, W. ; Yamamoto, Y. S. ; Campbell, F. T. ; Pfefferkorn, R. ; Rounsaville, J. F. Ullmann’s Encyclopedia of Industrial Chemistry; Wiley-VCH, 1985.

[10] Meyers, R. A. Handbook of Petroleum Refining Processes, 4th ed.; McGraw-Hill, 2016.

[11] Meyers, R. A. Handbook of Petrochemicals Production Processes, 2nd ed.; McGraw-Hill Education, 2019.

[12] Hammarström L., Styring S. (2011). Proton-coupled electron transfer
of tyrosines in Photosystem
II and model systems for artificial photosynthesis: the role of a
redox-active link between catalyst and photosensitizer. Energy Environ. Sci..

[13] Gagliardi C. J., Vannucci A. K., Concepcion J. J., Chen Z. F., Meyer T. J. (2012). The role
of proton coupled electron transfer in water oxidation. Energy Environ. Sci..

[14] Tyburski R., Liu T. F., Glover S. D., Hammarström L. (2021). Proton-Coupled
Electron Transfer Guidelines, Fair and Square. J. Am. Chem. Soc..

[15] Lloyd, L. Handbook of Industrial Catalysts; Springer, 2011.

[16] Warburton R. E., Soudackov A. V., Hammes-Schiffer S. (2022). Theoretical Modeling of Electrochemical
Proton-Coupled Electron Transfer. Chem. Rev..

[17] Deshpande S., Greeley J. (2020). First-Principles Analysis
of Coverage, Ensemble, and
Solvation Effects on Selectivity Trends in NO Electroreduction on
Pt_3_Sn Alloys. ACS Catal..

[18] Miu E. V., McKone J. R., Mpourmpakis G. (2022). The Sensitivity
of Metal Oxide Electrocatalysis
to Bulk Hydrogen Intercalation: Hydrogen Evolution on Tungsten Oxide. J. Am. Chem. Soc..

[19] VanGelder L. E., Brennessel W. W., Matson E. M. (2018). Tuning the redox profiles of polyoxovanadate-alkoxide
clusters via heterometal installation: toward designer redox Reagents. Dalton Trans..

[20] Chen Q., Goshorn D. P., Scholes C. P., Tan X. L., Zubieta J. (1992). Coordination
compounds of polyoxovanadates with a hexametalate core. Chemical and
structural characterization of [V^v^
_6_O_13_[(OCH_2_)_3_C^R^]_2_]^2‑^, [V^v^
_6_O_11_(OH)_2_[(OCH_2_)_3_C^R^]_2_], [V^iv^
_4_V^v^
_2_O_9_(OH)_4_[(OCH_2_)_3_CR]_2_]^2‑^, and [V^iv^
_6_O_7_(OH)_6_]­(OCH_2_)_3_CR]_2_]^2‑^. J. Am. Chem. Soc..

[21] Day V. W., Klemperer W. G. (1985). Metal-Oxide
Chemistry in Solution - the Early Transition-Metal
Polyoxoanions. Science.

[22] Warren J. J., Tronic T. A., Mayer J. M. (2010). Thermochemistry
of Proton-Coupled
Electron Transfer Reagents and its Implications. Chem. Rev..

[23] Mayer J. M. (2011). Understanding
Hydrogen Atom Transfer: From Bond Strengths to Marcus Theory. Acc. Chem. Res..

[24] Hammes-Schiffer S. (2010). Introduction:
Proton-Coupled Electron Transfer. Chem. Rev..

[25] Mayer J. M. (2023). Bonds over
Electrons: Proton Coupled Electron Transfer at Solid-Solution Interfaces. J. Am. Chem. Soc..

[26] Fertig A. A., Brennessel W. W., McKone J. R., Matson E. M. (2021). Concerted Multiproton-Multielectron
Transfer for the Reduction of O_2_ to H_2_O with
a Polyoxovanadate Cluster. J. Am. Chem. Soc..

[27] Fertig A. A., Matson E. M. (2023). Connecting Thermodynamics and Kinetics
of Proton Coupled
Electron Transfer at Polyoxovanadate Surfaces Using the Marcus Cross
Relation. Inorg. Chem..

[28] Marcus R. A., Sutin N. (1985). Electron transfers in chemistry and
biology. Biochim. Biophys. Acta.

[29] Maeda K., Katano H., Osakai T., Himeno S., Saito A. (1995). Charge dependence
of one-electron redox potentials of Keggin-type heteropolyoxometalate
anions. J. Electroanal. Chem..

[30] Zhou Y., Bihl F., Bonnefont A., Boudon C., Ruhlmann L., Badets V. (2022). Selectivity and efficiency
of nitrite electroreduction
catalyzed by a series of Keggin polyoxometalates. J. Catal..

[31] Wilson S. M., Petel B. E., Schreiber E., Maiola M. L., Su P., Matson E. M., Laskin J. (2023). Electrochemical
and Structural Characterization
of Soft Landed Tungsten-Substituted Lindqvist Polyoxovanadate-Alkoxides. Chem.—Eur. J..

[32] Li F., VanGelder L. E., Brennessel W. W., Matson E. M. (2016). Self-Assembled,
Iron-Functionalized Polyoxovanadate Alkoxide Clusters. Inorg. Chem..

[33] Meyer R. L., Brennessel W. W., Matson E. M. (2018). Synthesis of a gallium-functionalized
polyoxovanadate-alkoxide cluster: Toward a general route for heterometal
installation. Polyhedron.

[34] Shiels D., Lu Z., Pascual-Borrás M., Cajiao N., Marinho T. V., Brennessel W. W., Neidig M. L., Errington R. J., Matson E. M. (2024). Vanadium Substitution
Dictates H Atom Uptake at Lindqvist-type
Polyoxotungstates. Inorg. Chem..

[35] Fernández J. A., López X., Poblet J. M. (2007). A DFT study on the effect of metal,
anion charge, heteroatom and structure upon the relative basicities
of polyoxoanions. J. Mol. Catal. A:Chem..

[36] Towarnicky A., Lu Z., Matson E. M., Mpourmpakis G. (2025). Morphology Effects on Free Energies
of Proton-Coupled Electron Transfer in Polyoxotungstates. Inorg. Chem..

[37] Wise C. F., Agarwal R. G., Mayer J. M. (2020). Determining
Proton-Coupled Standard
Potentials and X-H Bond Dissociation Free Energies in Nonaqueous Solvents
Using Open-Circuit Potential Measurements. J.
Am. Chem. Soc..

[38] Bordwell F. G., Liu W.-Z. (1996). Solvent Effects
on Homolytic Bond Dissociation Energies
of Hydroxylic Acids. J. Am. Chem. Soc..

[39] Cooney S. E., Fertig A. A., Buisch M. R., Brennessel W. W., Matson E. M. (2022). Coordination-induced bond weakening of water at the
surface of an oxygen-deficient polyoxovanadate cluster. Chem. Sci..

[40] Fertig A. A., Rabbani S. M. G., Koch M. D., Brennessel W. W., Miro P., Matson E. M. (2021). Physicochemical
implications of surface
alkylation of high-valent, Lindqvist-type polyoxovanadate-alkoxide
clusters. Nanoscale.

[41] Proe K. R., Towarnicky A., Fertig A., Lu Z., Mpourmpakis G., Matson E. M. (2024). Impact of Surface Ligand Identity and Density on the
Thermodynamics of H Atom Uptake at Polyoxovanadate-Alkoxide Surfaces. Inorg. Chem..

[42] Agarwal R. G., Kim H. J., Mayer J. M. (2021). Nanoparticle
O-H Bond Dissociation
Free Energies from Equilibrium Measurements of Cerium Oxide Colloids. J. Am. Chem. Soc..

[43] Bard, A. J. F. L. R. ; White, H. S. Electrochemical Methods: Fundamentals and Applications, 3rd ed.; Wiley, 2022.

[44] Fripiat J. J., Lambert J. F. (1989). Statistical Thermodynamics Approach to the Hydrogen
Intercalation Process in Transition-Metal Oxides (H-Bronzes). J. Phys. Chem..

[45] Hill, T. L. An Introduction to Statistical Thermodynamics; Addison-Wesley Publishing Company, Inc.: Reading, Massachusetts, USA, 1960.

[46] Cheula R., Maestri M., Mpourmpakis G. (2020). Modeling Morphology and Catalytic
Activity of Nanoparticle Ensembles Under Reaction Conditions. ACS Catal..

[47] Kwon H., Mpourmpakis G. (2023). Thermochemistry
of Highly Flexible Molecules for Thermal
Decomposition Analysis. J. Chem. Theory Comput..

[48] Sutton C., Levchenko S. V. (2020). First-Principles Atomistic Thermodynamics
and Configurational
Entropy. Front. Chem..

[49] Fleck M., Zagrovic B. (2019). Configurational Entropy
Components and Their Contribution
to Biomolecular Complex Formation. J. Chem.
Theory Comput..

[50] Chan L., Morris G. M., Hutchison G. R. (2021). Understanding
Conformational Entropy
in Small Molecules. J. Chem. Theory Comput..

[51] Meirovitch H. (2010). Methods for
calculating the absolute entropy and free energy of biological systems
based on ideas from polymer physics. J. Mol.
Recognit..

[52] Suárez D., Díaz N. (2015). Direct methods
for computing single-molecule entropies
from molecular simulations. Wiley Interdiscip.
Rev. Comput. Mol. Sci..

[53] Frisch, M. J. ; Gaussian 16 Rev. A.03: Wallingford, CT, 2016.

[54] Becke A. D. (1993). Density-functional
thermochemistry. III. The role of exact exchange. J. Chem. Phys..

[55] Dunning, T. H. J. ; Hay, P. J. Modern Theoretical Chemistry; Plenum: New York, 1977; Vol. 3, pp 1–28.

[56] Sheppard B. J. H., Shaver M. P., Pearson J. K. (2015). Assessment and Application of Density
Functional Theory for the Prediction of Structure and Reactivity of
Vanadium Complexes. J. Phys. Chem. A.

[57] Marenich A. V., Cramer C. J., Truhlar D. G. (2009). Universal Solvation
Model Based on
Solute Electron Density and on a Continuum Model of the Solvent Defined
by the Bulk Dielectric Constant and Atomic Surface Tensions. J. Phys. Chem. B.

[58] Ochterski, J. W. Thermochemistry in Gaussian; Gaussian, Inc., 2022.

[59] Stratmann R. E., Burant J. C., Scuseria G. E., Frisch M. J. (1997). Improving harmonic
vibrational frequencies calculations in density functional theory. J. Chem. Phys..

[60] Glendening, E. D. ; Reed, A. E. ; Carpenter, J. E. ; Weinhold, F. NBO Version 3.1; Theoretical Chemistry Institute: University of Wisconsin: Madison, 1990.

[61] Dennington, R. K. T. ; Millam, J. GaussView. Version 6.1.1; Semichem Inc.: Shawnee Mission, KS, 2019.

[62] Pitman, J. Probability; Springer New York: New York, NY, United States, 1993.

[63] McQuarrie, D. A. Statistical Mechanics; University Science Books, 2000.

[64] Panagiotopoulos, A. Z. , Draft material from ″Statistical Thermodynamics″. http://paros.princeton.edu/cbe422/StatMech.pdf, 2014 (accessed Dec 7, 2025).

[65] Mori T. (2016). Macrostate
equivalence of two general ensembles and specific relative entropies. Phys. Rev. E.

[66] Pathria, R. K. ; Beale, P. D. Statistical Mechanics, 3rd ed.; Elsevier Ltd., 2011.

[67] Touchette H., Ellis R. S., Turkington B. (2004). An introduction to the thermodynamic
and macrostate levels of nonequivalent ensembles. Phys. A.

[68] Shell, M. S. Thermodynamics and Statistical Mechanics: An Integrated Approach; Cambridge University Press: Cambridge, 2015.

[69] Campa A., Dauxois T., Ruffo S. (2009). Statistical
mechanics and dynamics
of solvable models with long-range interactions. Phys. Rep..

[70] Seabold, S. ; Perktold, J. Statsmodels: Econometric and statistical modeling with python; Proceedings of the 9th Python in Science Conference: 2010.

[71] Sadakane M., Steckhan E. (1998). Electrochemical Properties
of Polyoxometalates as Electrocatalysts. Chem.
Rev..

[72] Chakraborty S., Schreiber E., Sanchez-Lievanos K. R., Tariq M., Brennessel W. W., Knowles K. E., Matson E. M. (2021). Modelling local structural and electronic
consequences of proton and hydrogen-atom uptake in VO_2_ with
polyoxovanadate clusters. Chem. Sci..

[73] Foresman, J. B. ; Frisch, A. E. Exploring Chemistry with Electronic Structure Methods, 3rd ed.; Gaussian, Inc., 2015.

[74] Jacobsen R. L., Johnson R. D., Irikura K. K., Kacker R. N. (2013). Anharmonic
Vibrational Frequency Calculations Are Not Worthwhile for Small Basis
Sets. J. Chem. Theory Comput..

[75] Slade, G. ; Blath, J. ; Imkeller, P. The self-avoiding walk: A brief survey Surveys in Stochastic Processes, Proceedings of the 33rd SPA Conference, Berlin, 2009, 181–199.

[76] Hait D., Head-Gordon M. (2018). Delocalization Errors in Density Functional Theory
Are Essentially Quadratic in Fractional Occupation Number. J. Phys. Chem. Lett..

[77] Miu E. V., Mpourmpakis G., McKone J. R. (2020). Predicting the Energetics of Hydrogen
Intercalation in Metal Oxides Using Acid-Base Properties. ACS Appl. Mater. Interfaces.

[78] Warburton R. E., Mayer J. M., Hammes-Schiffer S. (2021). Proton-Coupled Defects Impact O-H
Bond Dissociation Free Energies on Metal Oxide Surfaces. J. Phys. Chem. Lett..

