# Supplementary material for: Ensemble Effects on Hydroxide Bond Dissociation Free Energies in Polyoxovanadate Clusters
Source: J Phys Chem A. 2026 Feb 9;130(7):1433–46. doi: 10.1021/acs.jpca.5c04885 (PMC12927029; doi:10.1021/acs.jpca.5c04885)
Supplement: Supplementary file 1 [file jp5c04885_si_001.pdf]

Supporting Information for

Ensemble Effects on Hydroxide Bond Dissociation Free Energies in Polyoxovanadate Clusters

Andreas Towarnicky[1], John N. El Berch[1], and Giannis Mpourmpakis[1,2,*]

[1]Department of Chemical Engineering, University of Pittsburgh, Pittsburgh, PA 15261 USA

[2]School of Chemical Engineering, National Technical University of Athens (NTUA), Athens, GR-15780, Greece

*Corresponding author email address: gmpourmp@pitt.edu

Supporting Information Table of Contents

Figure S1. Relative energies for different POM spin states S3

Figure S2. Illustration of different O–H binding dihedral orientations S3

Table S1. Combinatorial numbers of distinct configurations, relevant spin states, and required calculations S4

Figure S3. Alternate counterions and coordinations considered S5

Figure S4. Illustration of differences and connections between ensemble frameworks S6

Figure S5. Experimental BDFE(O–H)$_{avg}$ for each POM vs. the number of V^{IV} centers S7

Figure S6. Cumulative energies of hydrogenation for POMs 2 and 3 ..... S7

Figure S7. Surface termination of V_2O_5 ..... S8

Figure S8. Comparison of POM 1 BDFE(O–H) with and without variable counterion positions S8

Figure S9: Contributions to BDFE(O–H) values of DFT electronic and thermal components S9

Figure S10. DFT calculated $O_B^{1\varphi}$ ensemble distributions for POM 1 ..... S10

Figure S11. DFT calculated $O_B^{1\varphi}$ ensemble distributions for POM 2 ..... S10

Figure S12. DFT calculated $O_B^{1\varphi}$ ensemble distributions for POM 3 ..... S11

Figure S13. Example graphical representation of possible H-binding configurations S11

Figure S14. Example enumeration of Q_{NS} for POM 1 with 3H, via graphical representations S12

Figure S15. S^{Config} and ΔG^{BA} impacts on BDFE(O–H) for POMs 1 and 2 ..... S13

Figure S16. S^{Config} and ΔG^{BA} impacts on POM 3 BDFE(O–H) S13

Figure S17. Impact of increasing ensemble consideration on BDFE(O–H) for POMs 1 and 2 ..... S14

Table S2. S^{Config} impacts on BDFE(O–H) for ceria nanoparticles investigated by Agarwal, Kim, and Mayer[1] S15

Figure S18. Comparison of S^{Config} impacts for different surface/metal ratios of Ce nanoparticles[1] and POMs S15

Figure S19. Bilinear modelling parity of BDFE(O–H) from DFT and DFT + TS^{Config} S16

Figure S20. Leave-out-one-point bilinear model parity of BDFE(O–H) from DFT and DFT + TS^{Config} S16

Figure S21. Leave-out-one-cluster bilinear model parity of BDFE(O–H) from DFT and DFT + TS^{Config} S17

Figure S22. Cross-validated bilinear model parity for BDFE(O–H)^{Ensemble} S17

Example S1. Linear Modelling of BDFE(O–H) via Slope and Endpoints, Equation S1 ..... S18

Figure S23. Experimental parity and bilinear model performance with BDFE(O–H)$_{avg}^{Linear}$ estimation S19

Figure S24. Bilinear model results from inputs per DFT and DFT + TS^{Config} S19

References S20

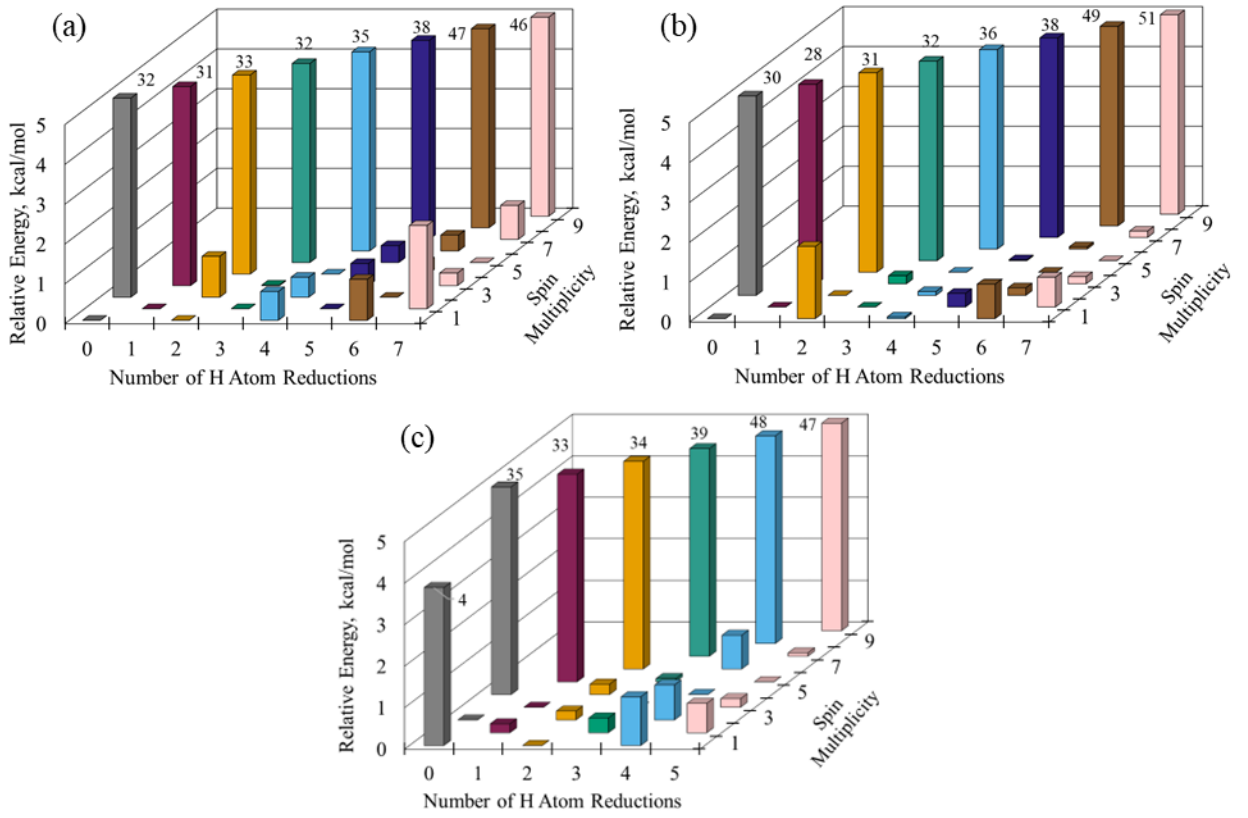

Figure S1: Relative free energies for different POM spin states, relative to the lowest energy state with the same number of H atom reductions, for (a) POM 1, (b) POM 2, and (c) POM 3. With each 2H reduction, an incremental spin state becomes near-isoenergetic with the lowest energy state. Here the y-axis is limited in order to emphasize the small differences between the near-isoenergetic spin states; higher spin energies above the y-axis maximum are labeled with their values. Changes in counterions did not significantly change spin stabilization trends.

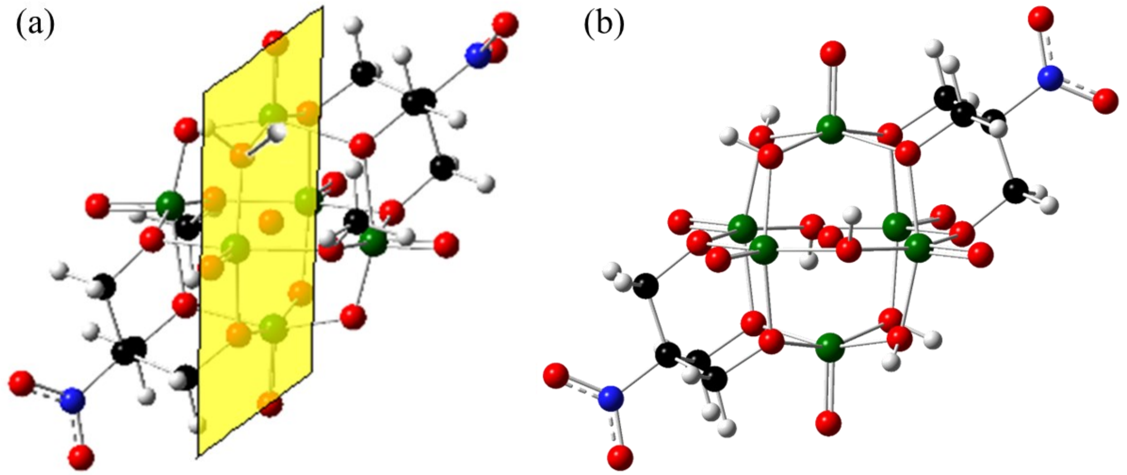

Figure S2: Illustration of O–H binding orientations, (a) POM 3 showing both possible H-binding orientations on the same O_B, with different dihedral angles with respect to the O=V–O plane (yellow shaded parallelogram), and (b) 6H on POM 1 adopting a thermodynamically preferable configuration without adjacent O–H steric hindrance, where all O–H appear to rotate about the cluster in the same direction. In (a) only one or the other of the O–H is present; 2H binding to the same O_B is not energetically accessible at ambient temperature (>30 kcal/mol higher in energy).

Table S1: Combinatorial numbers of distinct configurations for the $Q(O_B^{1\varphi})$ and $Q(O_B^{2\varphi})$ sets, numbers of near-isoenergetic spin states for each degree of H atom reduction, and the number of DFT geometry optimizations that would be required to resolve all $Q(O_B^{2\varphi})$ configurations and relevant spin states. In contrast, in the two right-most columns are the numbers of non-steric configurations (Q_{NS}) and the minimal number of DFT calculations required with the identified models (*vide infra*).

	Number of O–H on POM	$Q(O_B^{1\varphi})$	$Q(O_B^{2\varphi})$	Number of Relevant Spin States, #S	$\#S \times Q(O_B^{2\varphi})$ Required Calculations	Q_{NS}	$\#S \times Q(O_B^{1\varphi})$ Linear Model Required Calculations
POM 1 or 2	0	1	1	1	1	1	1
	1	6	12	1	12	12	6
	2	15	60	2	120	54	n/a
	3	20	160	2	320	112	n/a
	4	15	240	3	720	96	n/a
	5	6	192	3	576	36	18
	6	1	64	4	256	2	4
					Total: 2,005		

	Number of O–H on POM	$Q(O_B^{1\varphi})$	$Q(O_B^{2\varphi})$	Number of Relevant Spin States, #S	$\#S \times Q(O_B^{2\varphi})$ Required Calculations	Q_{NS}	$\#S \times Q(O_B^{1\varphi})$ Linear Model Required Calculations
POM 3	0	1	1	2	2	1	2
	1	4	8	2	16	6	8
	2	6	24	3	72	11	n/a
	3	4	32	3	96	6	12
	4	1	16	4	64	1	4
					Total: 250		

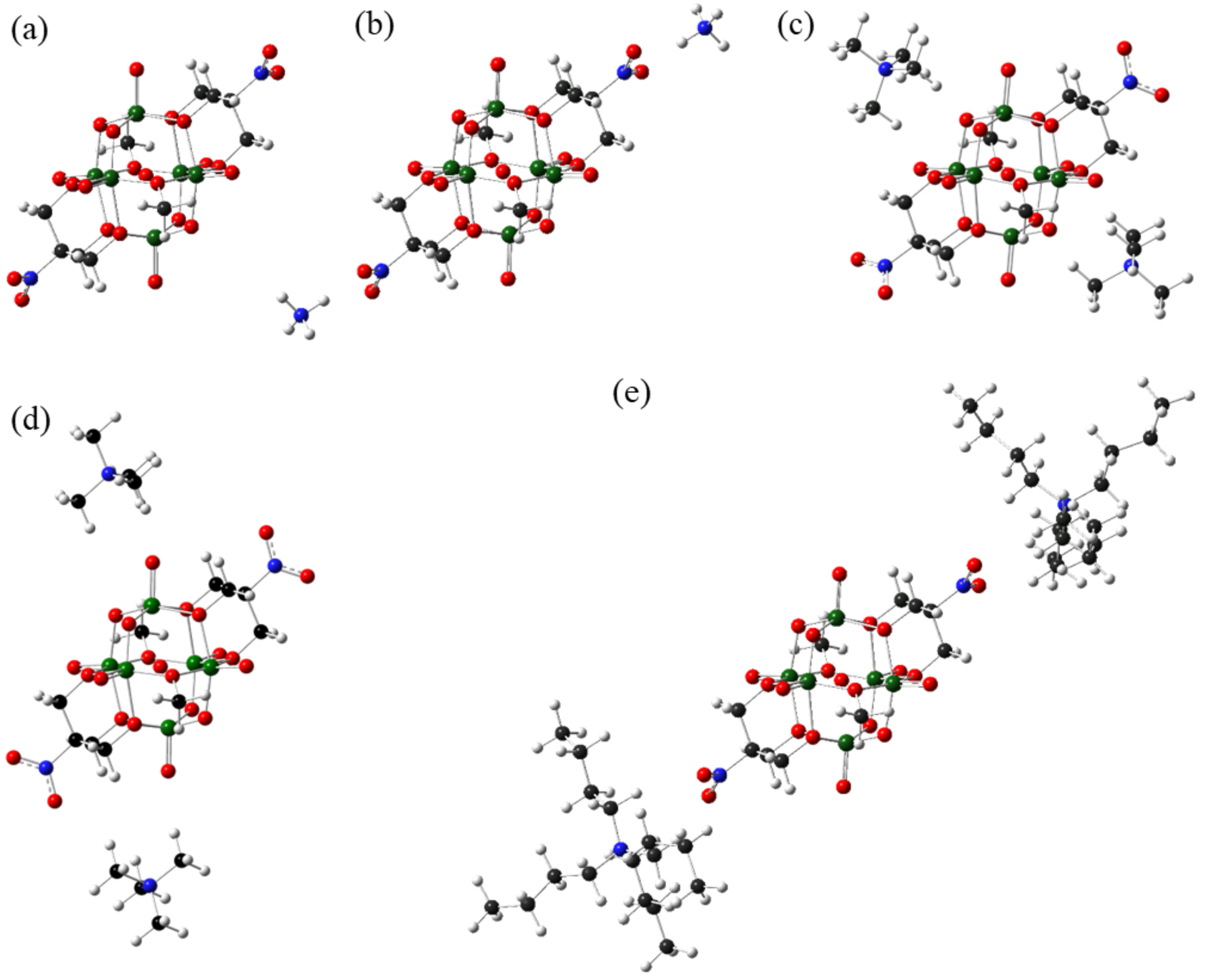

Figure S3: DFT geometrically optimized structure of POM 3 with different counterions and coordinations, (a) $2e^{-}$ in lieu of counterions, (b) $2NH_4^+$ counterions, (c) $2Me_4N^+$ coordinating to O_B atoms, (d) $2Me_4N^+$ coordinating to O_T atoms, and (e) 2 t-butyl$_4$N$^+$ counterions. Select combinations with $2Me_4N^+$ coordinating separately to $1O_T$ and $1O_B$ were also considered. Counterion identity was not found to have a significant effect on BDFE(O–H) trends.

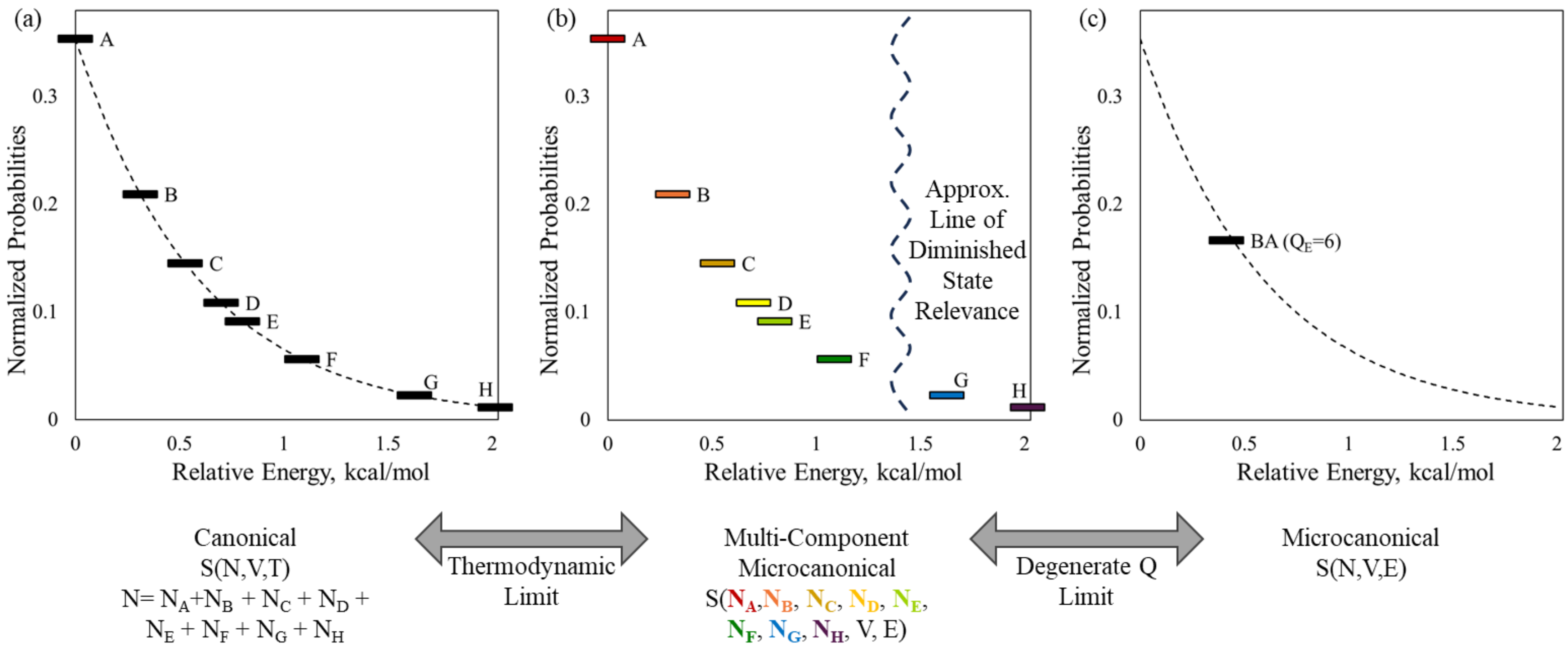

Figure S4: Illustration of differences and connections between ensemble frameworks for a solution of N POMs in acetonitrile for (a) canonical, (b) multi-component microcanonical (MC_Micro), and (c) microcanonical ensembles. In this figure the points labeled A-H represent different configurations, similar to ones that may be found on POM 3. BA stands for Boltzmann Average. In (b) N remains = $N_A + N_B + N_C + N_D + N_E + N_F + N_G + N_H$. Thus N is constant between (a) and (b) by definition. V is constant across all three ensembles. The T and E of (a) and (b) are both equivalent at the thermodynamic limit. To maintain equal N and equal E between (c) and (b), while realizing identical microcanonical particles, requires that each of the N particles in (c) must have energy equal to the Boltzmann average energy of (b). The degeneracy of the identical microcanonical particles is determined in the limit where relative energies between similar (e.g. non-steric) configurations vanish, while higher energy configurations are excluded.

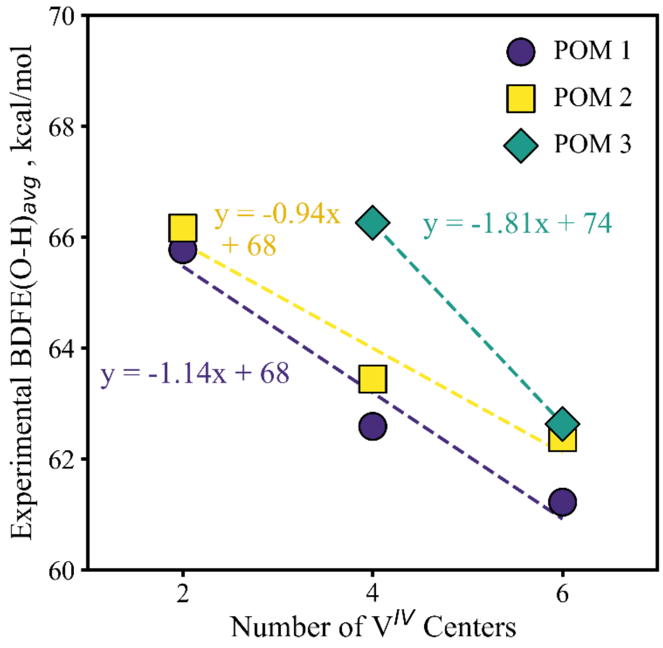

Figure S5: Experimental BDFE(O–H)$_{avg}$ for each POM vs. the number of V^{IV} centers. The number of V^{IV} centers is per the second O–H of the BDFE(O–H)$_{avg}$. Trends are plotted vs. V^{IV} centers to show the values at the same cluster oxidation states.

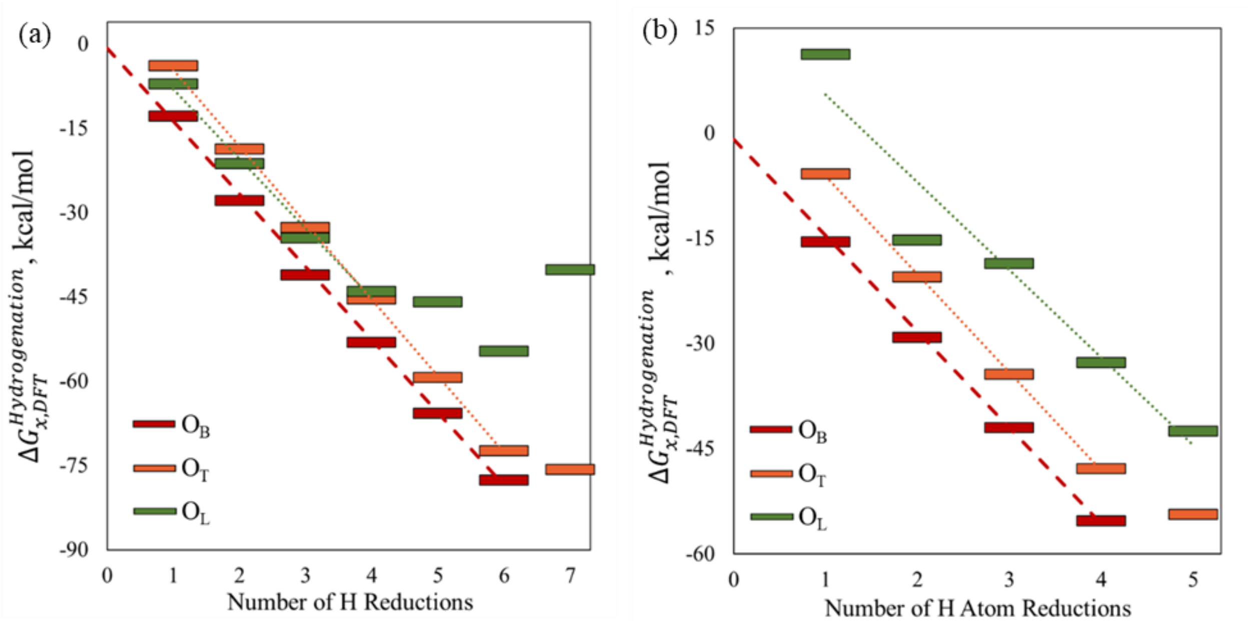

Figure S6: Cumulative energies of hydrogenation per Eqn. 5 for (a) POM 2 and (b) POM 3. No O_N sites exist in (a) and were not considered in (b) based on the results for POM 1 (main text Figure 3). O_B are consistently most favored.

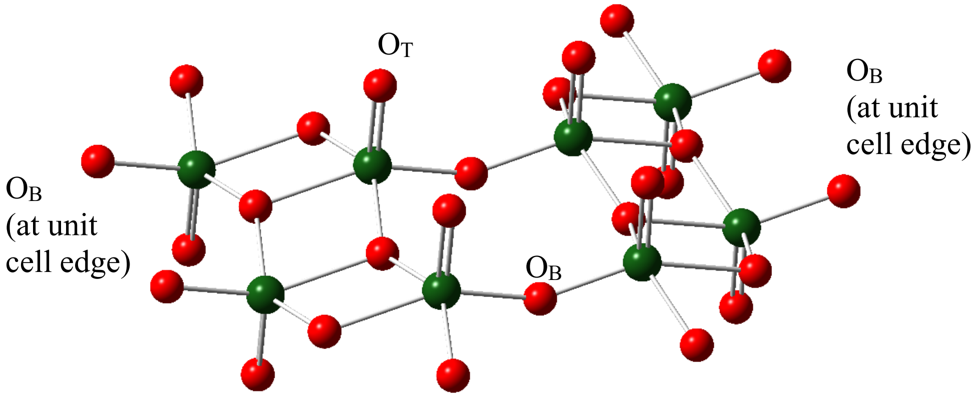

Figure S7: Surface termination of V_2O_5. Upper and lower terminations both possess O_B, O_T, and thrice-bonded O (same coordination number as O_L). Atomic positions are per bulk structure[2].

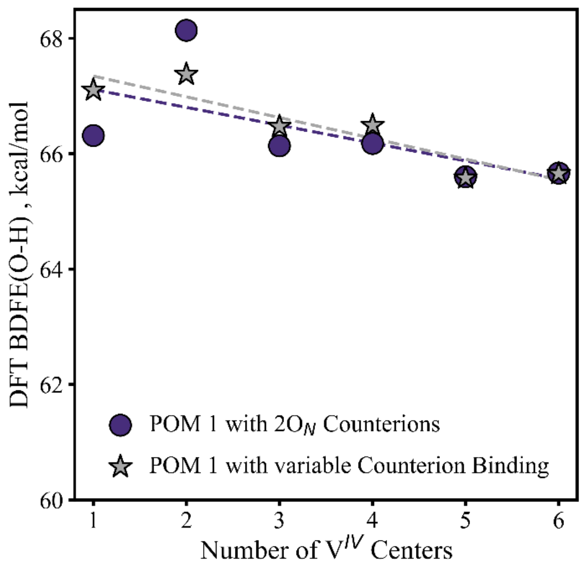

Figure S8: Comparison of POM 1 BDFE(O–H) with and without variable positions of counterion coordination across a series of 6H-atom reductions. The dark blue circles represent counterion coordinations as illustrated in main text Figure 1, and the grey stars consider additional possible coordinations as shown in Figure S3c, S3d, and combinations thereof. The lower 1st BDFE(O–H) is somewhat more balanced with the higher 2nd, perhaps due to a different counterion coordination slightly better stabilizing the asymmetric first H addition, but otherwise the impacts appear negligible.

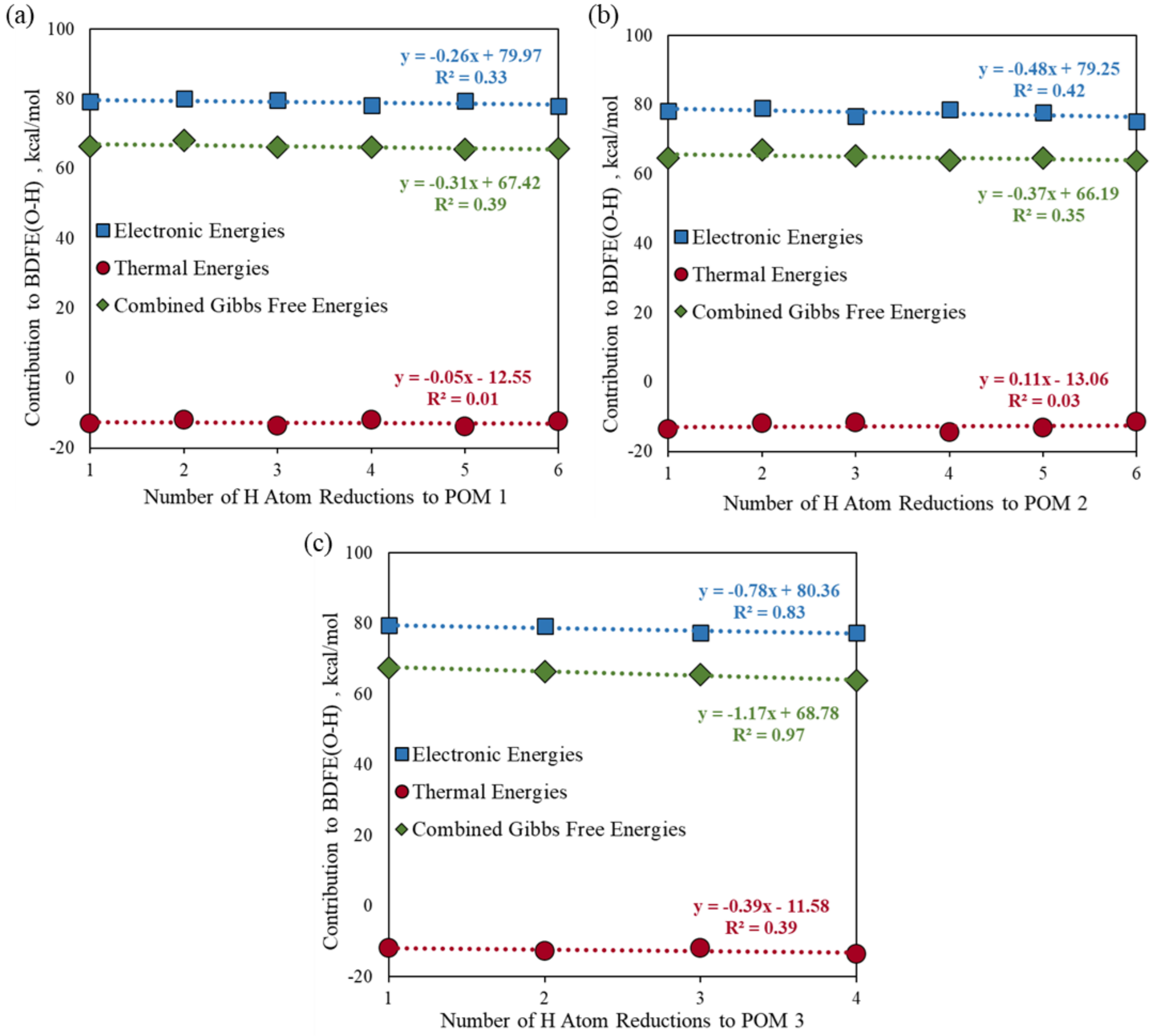

Figure S9: Contributions to BDFE(O–H) values of DFT electronic and thermal components, for (a) POM 1, (b) POM 2, and (c) POM 3.

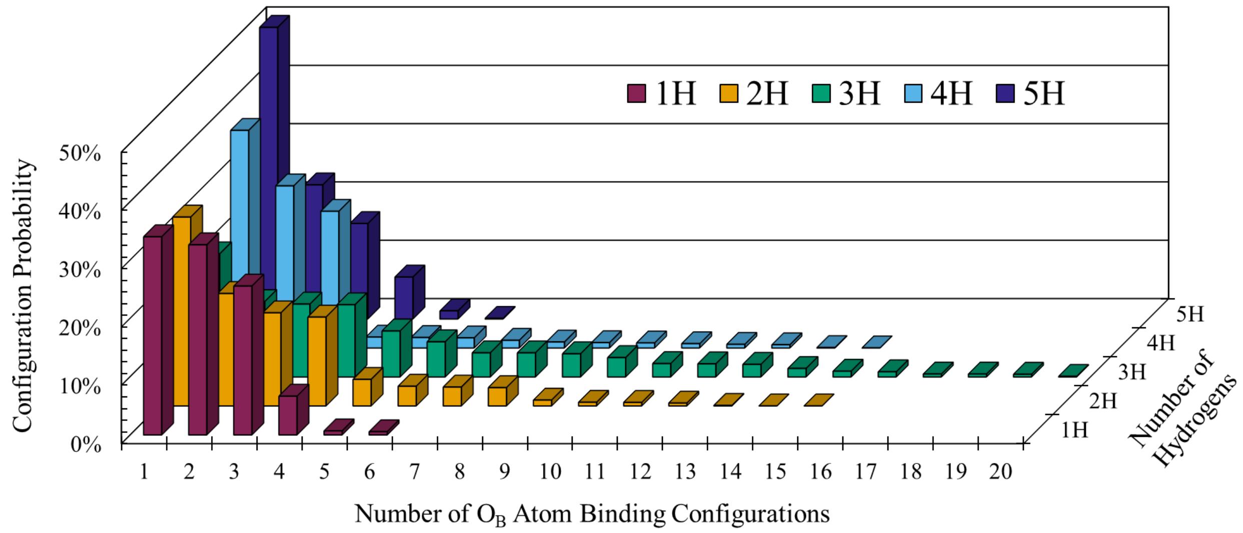

Figure S10: Normalized POM 1 ensemble distributions from DFT calculated free energies of the $O_B^{1\varphi}$ set. Probabilities were determined via Boltzmann statistics (main text Eqn. 7) and normalized on the basis of the $O_B^{1\varphi}$ numbers of configurations (Table S1, 144 DFT calculations in total). Resolution of the full $O_B^{2\varphi}$ set for this POM was prohibitive (at least 2,005 calculations).

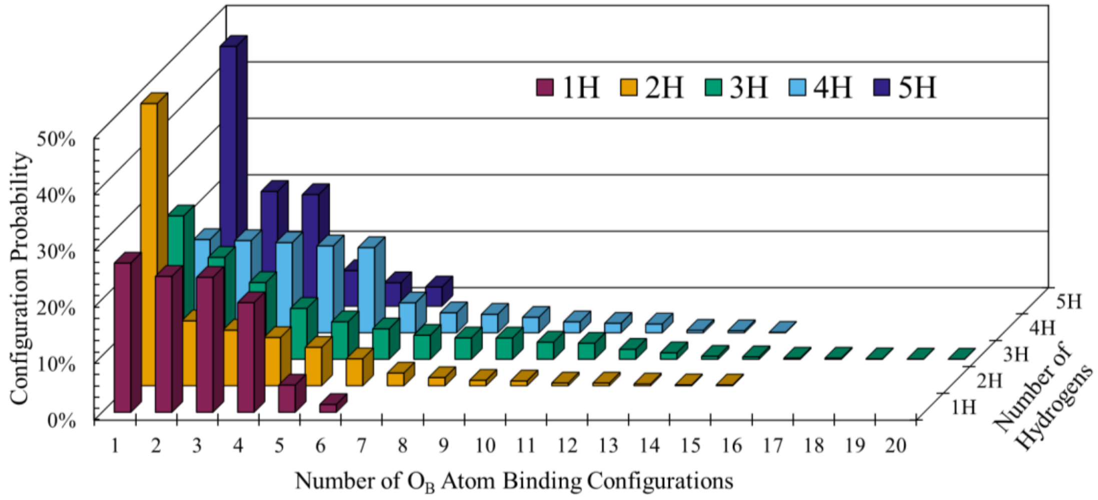

Figure S11: Normalized POM 2 ensemble distributions from DFT calculated free energies of the $O_B^{1\varphi}$ set. Probabilities were determined via Boltzmann statistics (main text Eqn. 7) and normalized on the basis of the $O_B^{1\varphi}$ numbers of configurations (Table S1, 144 DFT calculations in total). Resolution of the full $O_B^{2\varphi}$ set for this POM was prohibitive (at least 2,005 calculations).

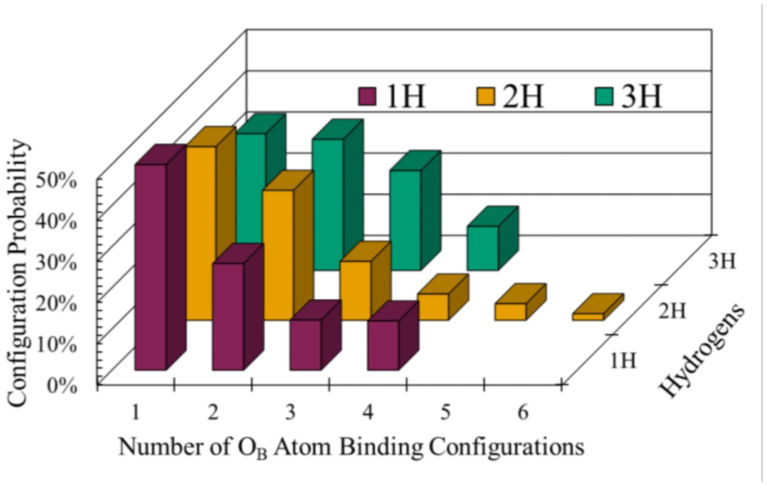

Figure S12: Normalized POM 3 ensemble distributions from DFT calculated free energies of the $O_B^{1\varphi}$ set. Probabilities were determined via Boltzmann statistics (main text Eqn. 7) and normalized on the basis of the $O_B^{1\varphi}$ numbers of configurations (Table S1, 44 DFT calculations in total). Figure S12 may be compared with main text Figure 4, wherein the full $O_B^{2\varphi}$ set for this POM was resolved (Table S1, 250 calculations in total).

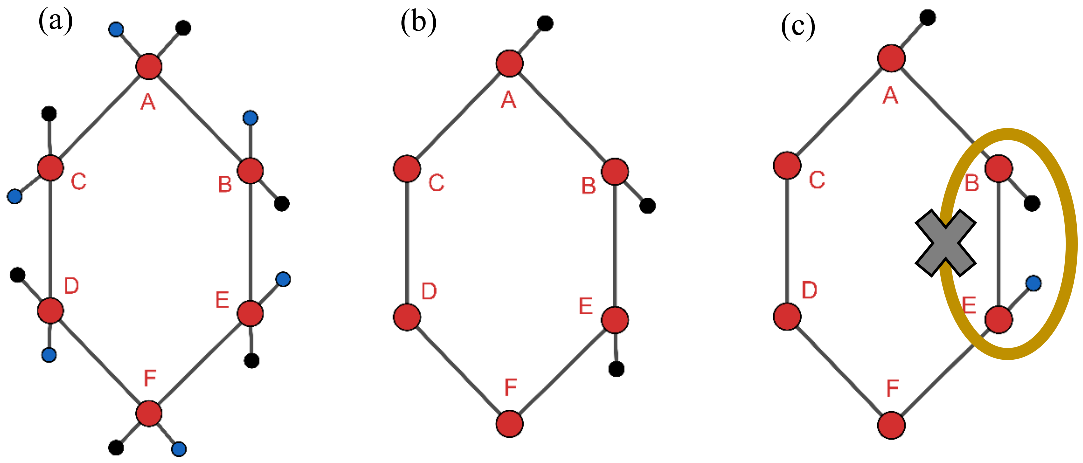

Figure S13: Graphical representations of (a) all possible O–H binding sites, with black and blue circles representing H atoms that would form different O=V–O–H dihedral angles, (b) a non-sterically hindered configuration from one combination of 3 specific H-binding sites, and (c) a sterically hindered configuration from another 3H combination.

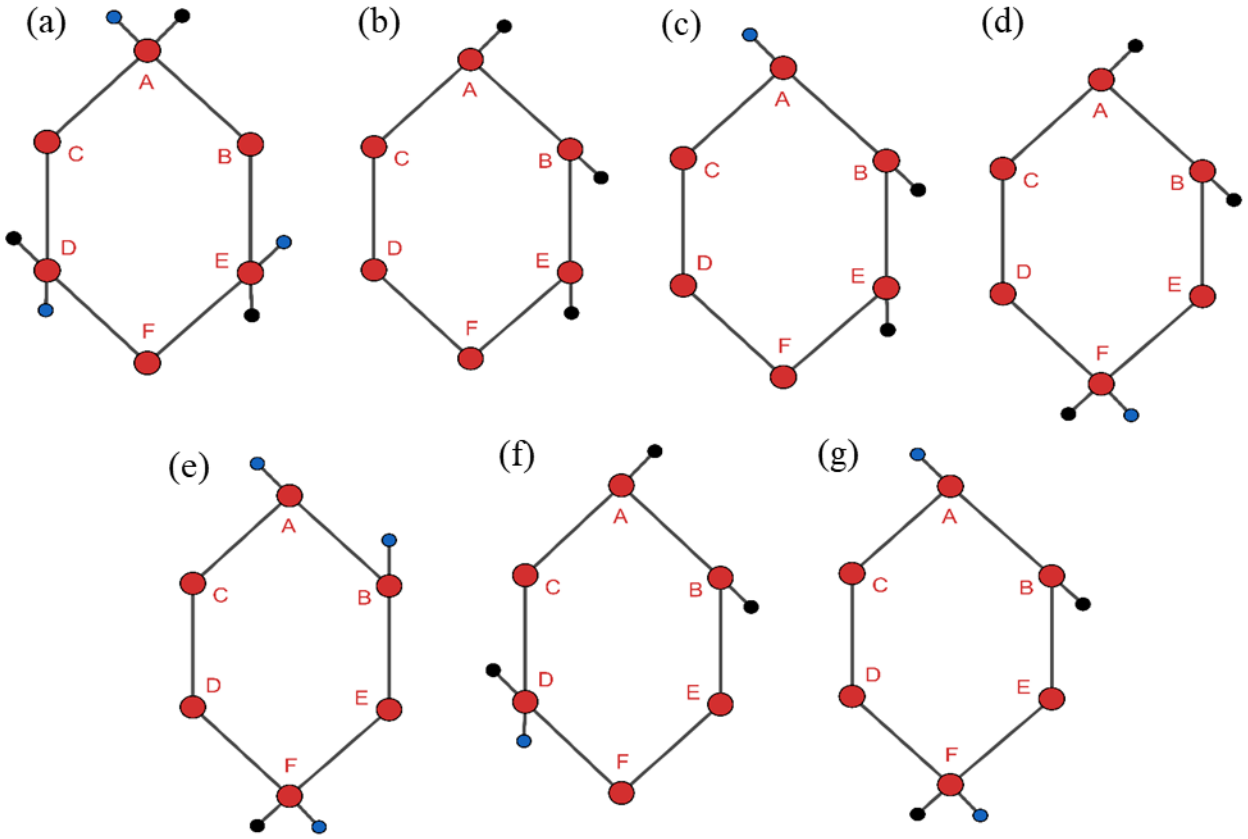

Figure S14: Graphical representations of non-sterically hindered configurations for POMs 1 and/or 2, with 3 H-atom reductions. Distinct O_B are labeled ABCDEF and ortho, meta, para terminology is used to describe relative positions: (a) displays both possible dihedral angle position for 3 meta O_B H-bindings, (b) shows 3 adjacent H-bindings (1 ortho, 1 meta), (c) specifically distinguishes the 2nd possible orientation for the O–H at the O_B represented by A, (d) illustrates the 1 ortho and 1 para H-bindings, with 2 dihedral orientations possible for the para-located O–H, (e) is the AB inversion of the (d) dihedrals, (f) illustrates 1-ortho and 2 possible H-bindings in the opposite meta direction, and (g) is the different dihedral orientation of (d) and/or (e). Alternatively, sterically hindered graphical representations and the total number of configurations may be determined by the same method, which aligns with main text Eqn. 28. Configurational enumeration per the above representations follows below (capital "F" etc. refer to the lettered sites on each representation):

$$(a) = 2^3 \times 2\ distinct\ positional\ rotations = 16$$
$$(b) = 6\ rotations\ \times 2\ inverted\ orientations = 12$$
$$(c) = 6\ rotations\ \times 2\ inverted\ orientations = 12$$
$$(d) = 6\ rotations\ \times 2\ orientation\ possibilities\ for\ F = 12$$
$$(e) = 6\ rotations\ \times 2\ orientations\ for\ F = 12$$
$$(f) = 6\ rotation\ \times 2\ D\ orientations\ \times 2\ AB\ inversion = 24$$
$$(g) = 6\ rotations\ \times 2\ F\ orientations \times 2\ DF\ mirror = 24$$
$$Total\ relevant\ configurations\ for\ POM_{3H}^{1\ or\ 2} = 16 + 12 + 12 + 12 + 12 + 24 + 24 = 112$$

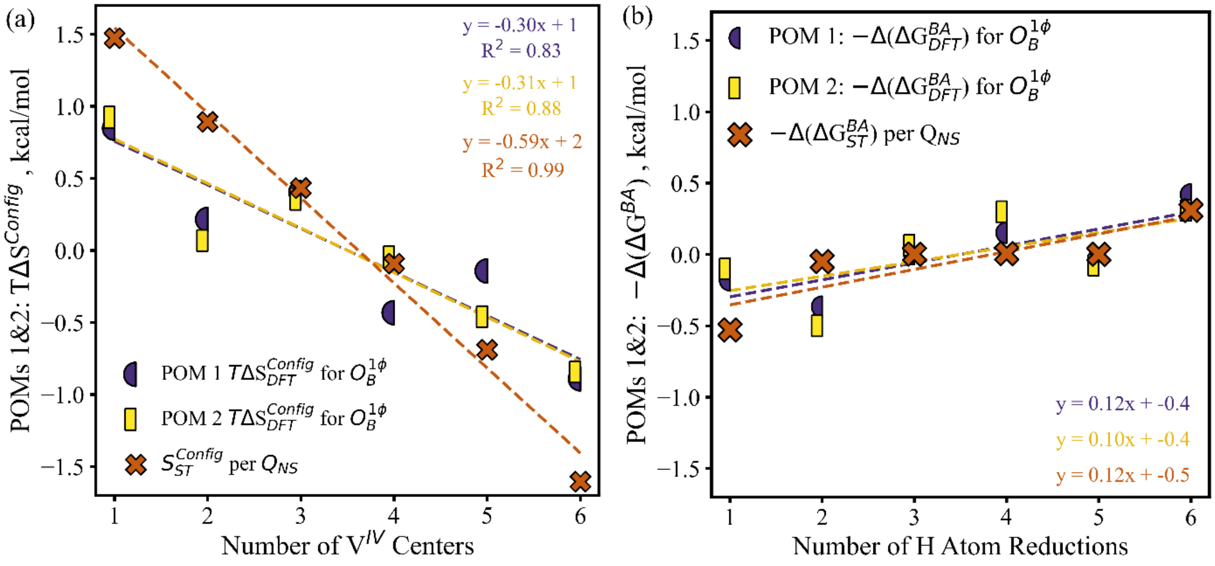

Figure S15: Impact on POM 1 and 2 BDFE(O–H) of considering (a) S^{Config} and (b) ΔG^{BA} . Half-filled markers for DFT-derived values are used to emphasize that they are based on the smaller $O_B^{1\varphi}$ ensembles.

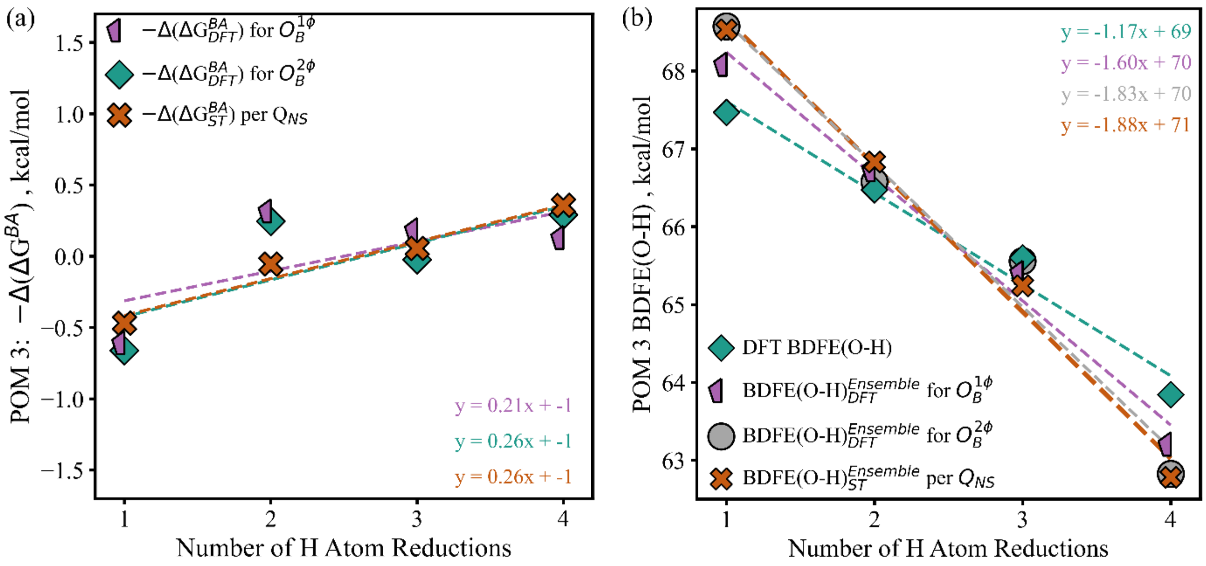

Figure S16: (a) Impact of considering ΔG^{BA} on BDFE(O–H) for POM 3, ΔG_{ST}^{BA} vs. ΔG_{DFT}^{BA}, and (b) POM 3 BDFE(O–H) with increasing consideration of S_{Config} and ΔG^{BA} effects, including alignment of POM 3 BDFE(O–H)^{Ensemble} calculated with DFT vs. ST methods. Half-filled markers for DFT-derived values are used to emphasize that they are based on the smaller $O_B^{1\varphi}$ ensembles. In (a) the remaining trend gap vs. experiments may be taken to be zero. With (b) the comparable experimental BDFE(O–H) slope is -1.81, with some offset in absolute values (addressed separately in the main text); for clarity a separate line is not shown.

.

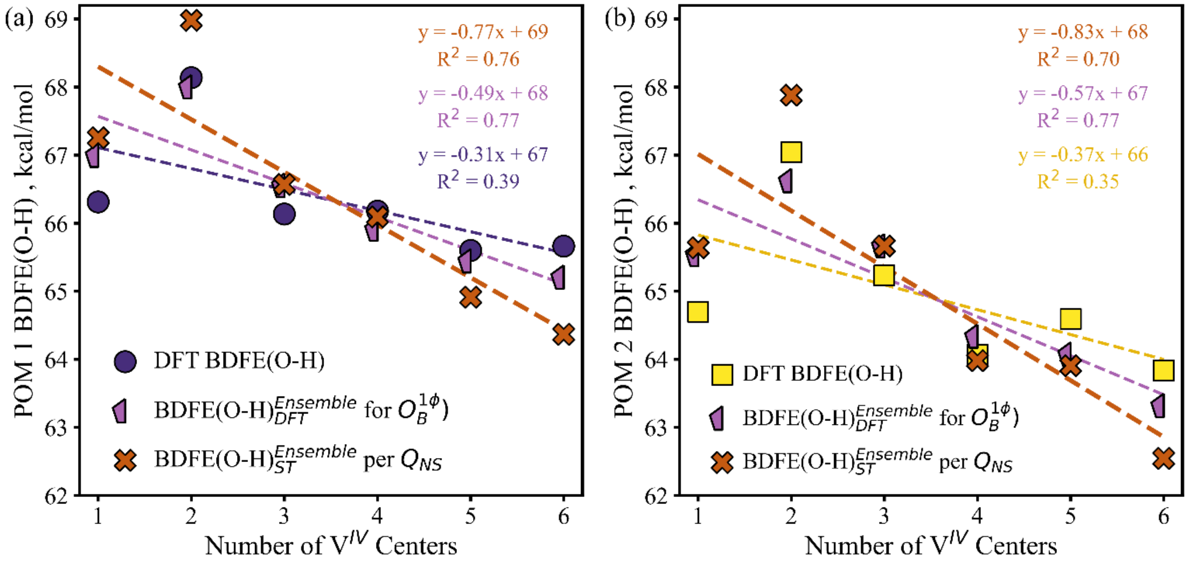

Figure S17: Impact on POMs 1 and 2 BDFE(O-H) from different possible determinations of S^{Config} and ΔG^{BA}. Half-filled markers for DFT-derived values are used to emphasize that they are based on the smaller $O_B^{1\varphi}$ ensembles. For comparison, the experimental slopes are -1.14 for POM 1, and -0.94 for POM 2 (units of kal/mol of BDFE(O–H) per H atom reduction); for clarity separate lines are not shown.

Table S2: Determination of the impact of considering $T\Delta S_{ST}^{Config}$ on the change in BDFE(O–H) across the domain of cerium reduction, for the hydrogenation of ceria nanoparticles that were investigated by Agarwal, Kim, and Mayer[1]. The number of configurations assumes 2 sites per $O_{Surface}$ atom, analogous to the present POM O_B. [a] The experimental BDFE(O–H) span is calculated for 0% to 100% Ce^{3+} (can compare to Figure 5 of [1]). [b] The impact across the series is approximated as 2 times the impact for $T\Delta S_{0H\rightarrow 1H}^{Config}$, consistent with our present POM findings (main text Figure 5).

Nanoparticle	Diameter, nm	Total # of Ce	# Surface Ce Sites	# Surface O Sites	# of H Atom Reductions	# of Configurations, $\#O_{Surface} \times 2$	$T\Delta S^{Config}$, 0H → 1H, kcal/mol	Experimental BDFE Span [a], kcal/mol	% of Trend Accounted for by Ensemble Effects [b]
Ce-1	1.8 ± 0.2	77	65	130	0	1	3.3	15.5	43%
					1	260			
Ce-2	1.9 ± 0.2	91	73	146	0	1	3.4	16.1	42%
					1	292			
Ce-L	4.0 ± 0.4	846	322	644	0	1	4.2	36.0	24%
					1	1288			

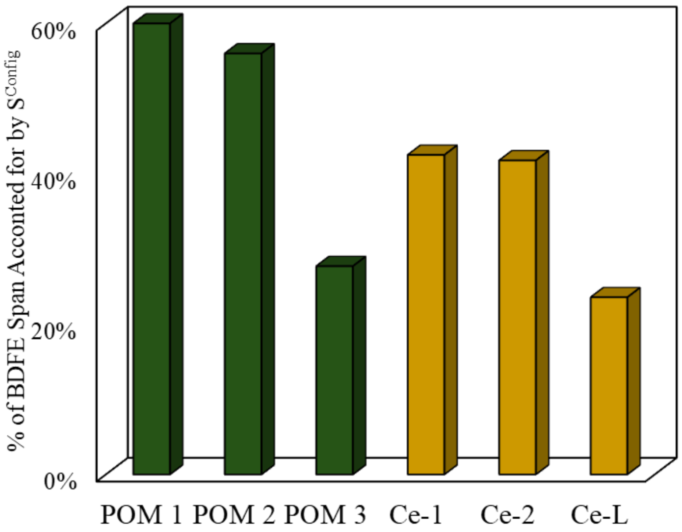

Figure S18: Percents of the POM and experimentally[1] measured ceria BDFE(O–H) spans accounted for by the $T\Delta S_{ST}^{Config}$ ensemble effect (Table S2).

Considering the different % impacts of S_{ST}^{Config} for the different particles: Agarwal, Kim, and Mayer[1] noted that increasing surface/volume ratios plays an important role for ceria nanoparticles in stabilizing Ce^{4+} reduction to Ce^{3+}. From the numbers of surface and total cerium (Table S2), the surface/volume ratios of Ce-1, Ce-2, and Ce-L are calculated as 0.84, 0.80, and 0.38, respectively. Aligning with this are the POM O_B/V ratios: 1.0 for POM 1 and 2, and 0.67 for POM 3. We also note that POM 3 has reduced S^{Config} impact (Figure 5) and increased ΔG^{BA} impact per H addition (Figures S15b and S16a) relative to POMs 1 and 2, directionally in line with the 4/6 O_B/V ratio and its inversion, respectively. However, these comparisons may be considered anecdotal.

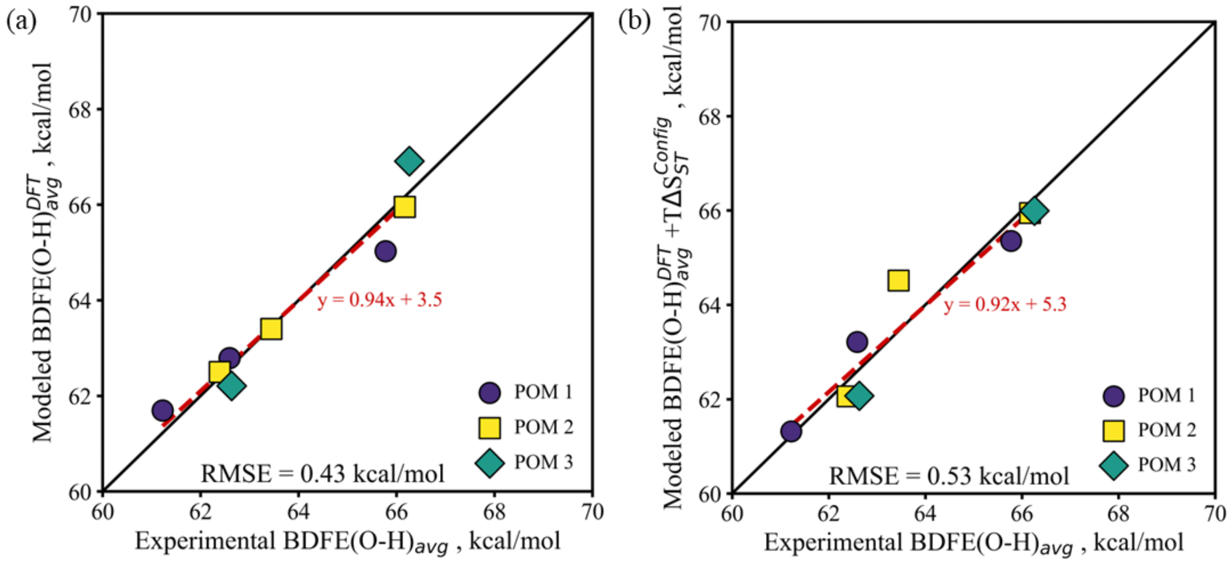

Figure S19: Parity results for bilinear modelling of (a) BDFE(O–H)$_{avg}^{DFT}$ (without ensemble effects) and (b) BDFE(O–H)$_{avg}^{DFT}$ + $T\Delta S_{ST}^{Config}$. In (a) the model has not captured the trend for all clusters; the 0.94 overall slope vs. parity has benefitted from cancelling errors in the POM 1 and POM 3 slopes. The lack of trend capture in (a) becomes even clearer with Figure S20a and S21a.

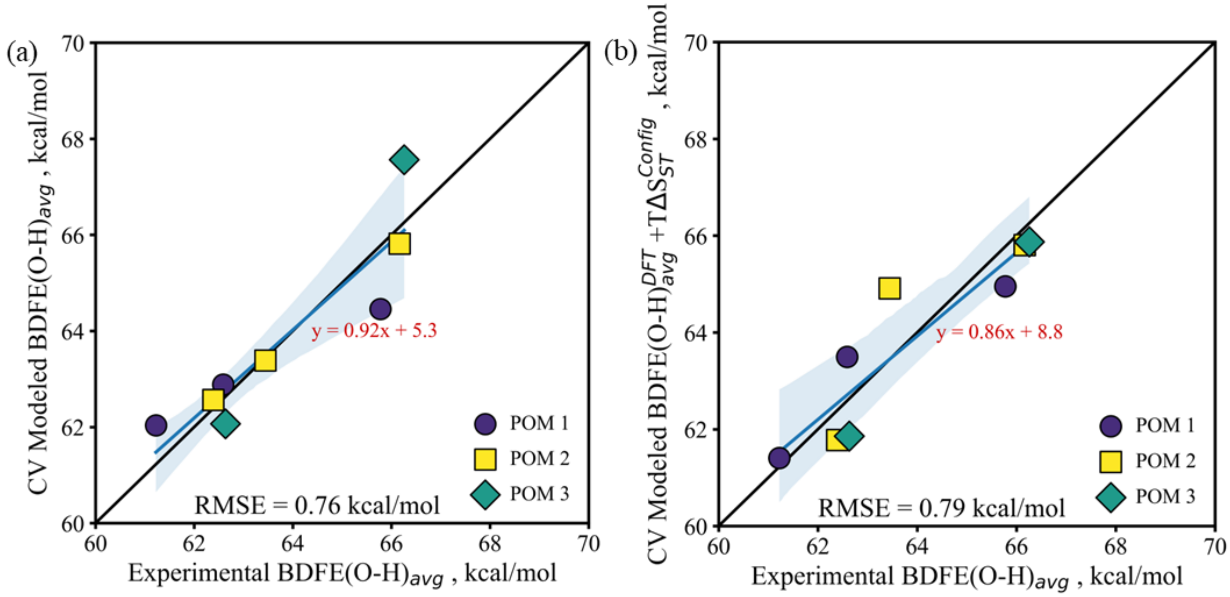

Figure S20: Leave-out-one-datapoint cross-validated parity results for bilinear modelling of (a) BDFE(O–H)$_{avg}^{DFT}$ (without ensemble effects) and (b) BDFE(O–H)$_{avg}^{DFT}$ + $T\Delta S_{ST}^{Config}$. In (a) the model has not captured the trend for all clusters; the 0.92 overall slope vs. parity has benefitted from cancelling errors in the POM 1 and POM 3 slopes.

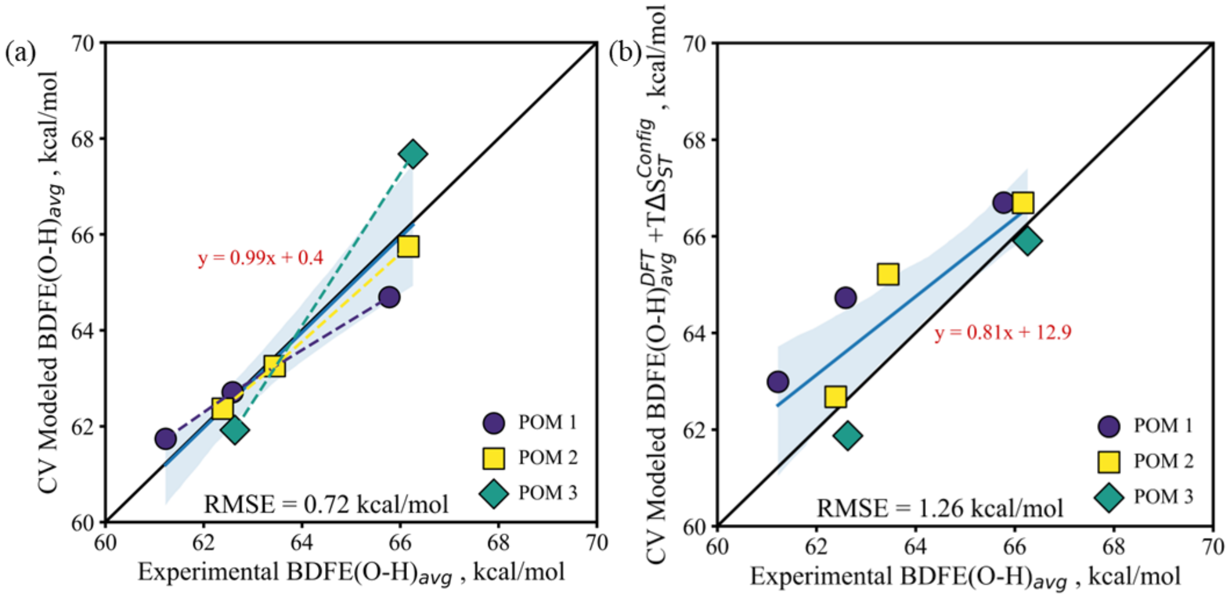

Figure S21: Leave-out-one-POM cross-validated parity results for bilinear modelling of (a) BDFE(O–H)$_{avg}^{DFT}$ (without ensemble effects) and (b) BDFE(O–H)$_{avg}^{DFT}$ + $T\Delta S_{ST}^{Config}$. In (a) the model has not captured the trend for all clusters; the 0.99 overall slope vs. parity has benefitted from cancelling errors in the POM 1 and POM 3 slopes.

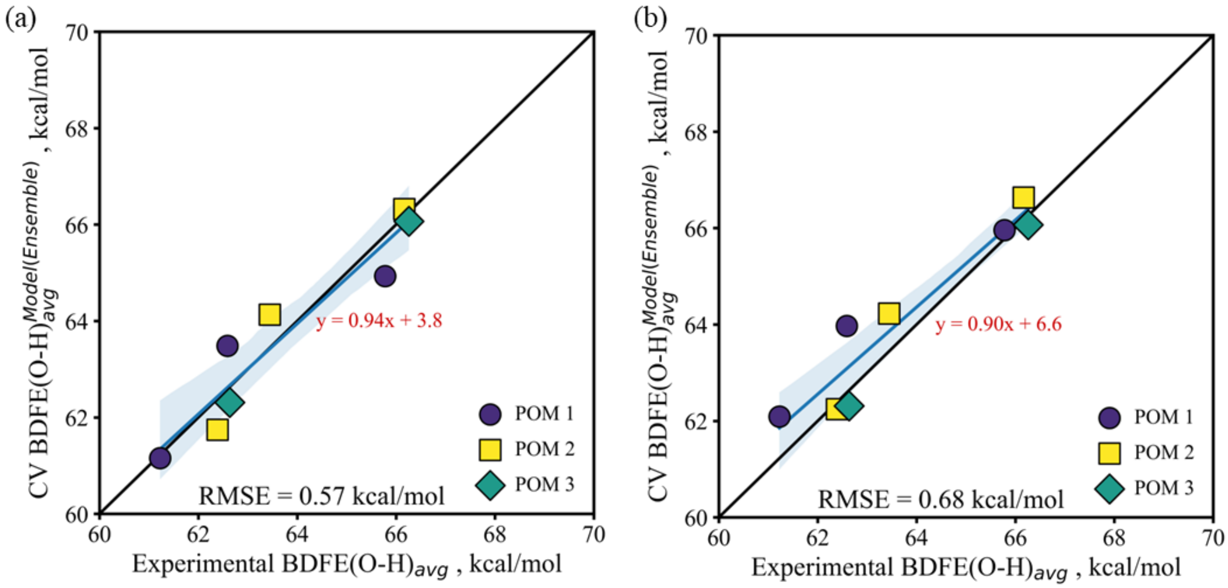

Figure S22: Cross-validated model results for BDFE(O–H)$_{avg}^{Ensemble}$ based on (a) leave-out-one-datapoint, and (b) leave-out-one-POM.

Example Linear Modelling of BDFE(O–H) via Slope and Endpoints:

The trend slope may be obtained by combining the slope of the S_{ST}^{Config} impact from statistical thermodynamic treatment per Q_{NS} used herein with the slope from DFT resolution of the first and last BDFE(O–H) for the $O_B^{1\varphi}$ set. Alternatively, the trend slope could be informed via experiment. Proceeding with an entirely theoretical approach, the $O_B^{1\varphi}$ set requires few H-binding configurations to be considered for these degrees of H-binding, and may be utilized as opposed to the $O_B^{2\varphi}$ set based on the preferred dihedral angle orientation of the fully H-bound cluster. The first and last BDFE(O–H) values inform the linear model's slope together with $\mathrm{T}\Delta S_x^{Config}$, and the absolute energies of the non- and fully- H-bound endpoints inform its average per their energetic span. Let z represent the total number of H-bindings to the POM. Mathematically:

$$BDFE(O-H)_x^{Linear} =$$

$$\frac{\left[G_{0H}^{POM} - G_{zH}^{POM} + zG^{H^\bullet}\right]}{z} + \frac{x}{z}\left[\mathrm{BDFE(O-H)}_{zH}^{DFT} - BDFE(O-H)_{1H}^{DFT}\right] + \mathrm{T}\Delta S_x^{Config}$$

Eqn. S1

The BDFE(O–H)^{Linear} developed from this approach align well with those calculated via DFT with S_{ST}^{Config} (Figure S23a vs. Figure 7a), and were further evaluated via treatment with the bilinear model of Eqn. 29. The bilinear model output from inputting the BDFE(O–H)^{Linear} achieved comparable parity vs. experimental values as if the intermediate configurations had been resolved via DFT (Figure S23b vs. Figure S19b).

For comparison, we also calculated the Eqn. 29 bilinear model output from inputting the BDFE(O–H)$_{avg}^{DFT}$, without ensemble effects (Figure S24a), and BDFE(O–H)$_{avg}^{DFT}$ + $T\Delta S_{ST}^{Config}$ (Figure 24b). From Figure S24 one may note the improvement in parity from (a) to (b), and further improvement to Figure 8a as more of the BDFE(O–H)$_{avg}^{Ensemble}$ components are included.

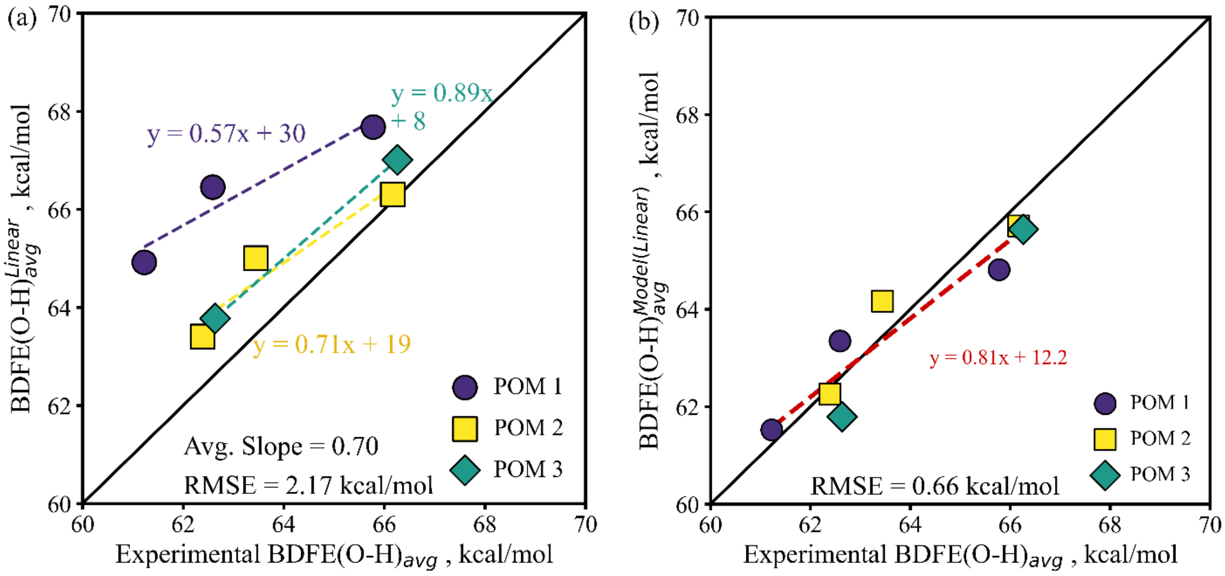

Figure S23: Parity vs. experimental values of (a) BDFE(O–H)$_{avg}^{Linear}$, and (b) bilinear model outputs with BDFE(O–H)$_{avg}^{Linear}$ inputs. The linear model results of (a) are comparable to those of main text Figure 7a.

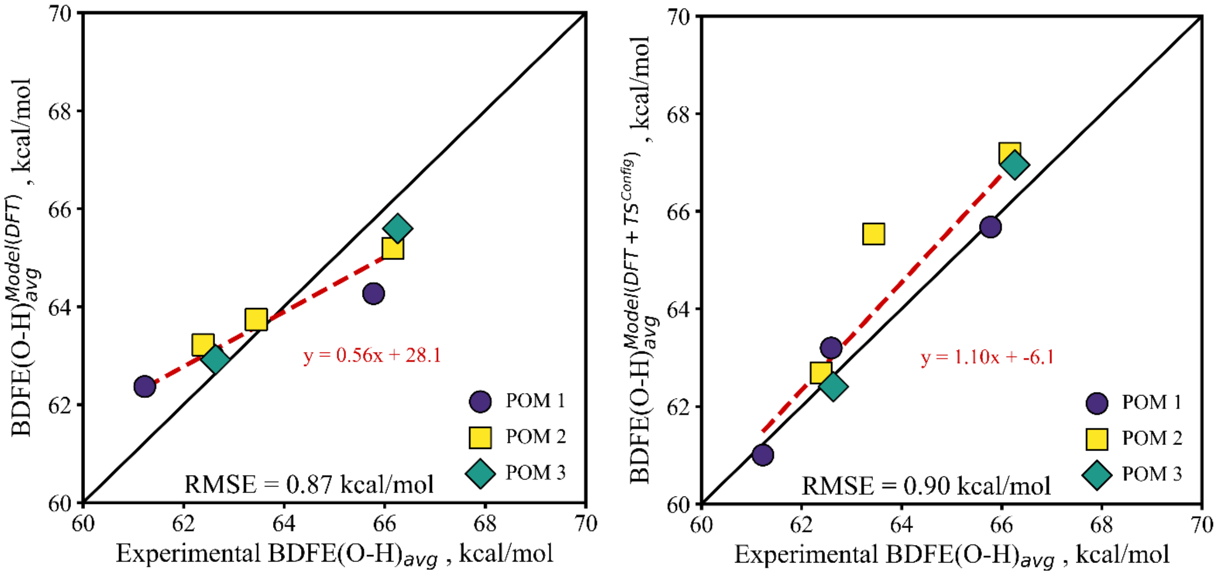

Figure S24: Bilinear model (Eqn. 29) results with inputs of (a) BDFE(O–H)$_{avg}^{DFT}$ (without ensemble effects) and (b) BDFE(O–H)$_{avg}^{DFT}$ $+ T\Delta S_{ST}^{Config}$.

SI References:

1. Agarwal, R. G.; Kim, H. J.; Mayer, J. M., Nanoparticle O-H Bond Dissociation Free Energies from Equilibrium Measurements of Cerium Oxide Colloids. *J Am Chem Soc* **2021**, *143*, 2896-2907.
2. Jain, A., et al., Commentary: The Materials Project: A Materials Genome Approach to Accelerating Materials Innovation. *Apl Mater* **2013**, *1*.
